# Olivetol induces a non-genotoxic nucleolar DNA damage response via membrane-dependent stress signaling

**DOI:** 10.1080/19491034.2026.2672818

**Published:** 2026-05-18

**Authors:** Dmitry A. Deriglazov, Nadezhda V. Petrova, Artem V. Luzhin, Nataliya G. Pavlenko, Victor V. Tatarskiy, Eugene P. Kazakov, Igor I. Kireev, Alexander V. Kurkin, Anton M. Novoselov, Yuri M. Efremov, Peter S. Timashev, Sergey V. Razin, Artem K. Velichko

**Affiliations:** a Department of Cellular Genomics, Institute of Gene Biology RAS, Moscow, Russia; b Laboratory of Molecular Oncobiology, Institute of Gene Biology RAS, Moscow, Russia; c A.N. Belozersky Institute of Physico-Chemical Biology, Lomonosov Moscow State University, Moscow, Russia; d Chemistry Department, Lomonosov Moscow State University, Moscow, Russia; e Institute for Regenerative Medicine, Sechenov First Moscow State Medical University, Moscow, Russia; f Biological Faculty, Lomonosov Moscow State University, Moscow, Russia; g Institute for Translational Medicine and Biotechnology, Sechenov First Moscow State Medical University, Moscow, Russia

**Keywords:** Olivetol, nucleolar DNA damage response, ribosomal DNA, R-loops, TOPBP1, Treacle, transcriptional conflict, hypotonic stress, plasma membrane organization, endoplasmic reticulum stress

## Abstract

Olivetol (5-pentylresorcinol) is a naturally occurring alkylresorcinol whose cellular mechanism remains poorly understood. Here, we show that olivetol induces a non-genotoxic nucleolar DNA damage response (n-DDR) in human cells. Although moderately cytotoxic, olivetol did not cause detectable genomic DNA double-strand breaks. Instead, it triggered γH2AX accumulation at ribosomal DNA (rDNA), recruitment of TOPBP1 to Treacle, nucleolar disorganization, and repression of ribosomal RNA synthesis. Mechanistically, olivetol closely phenocopied hypotonic stress, inducing a rapid and reversible n-DDR associated with antisense RNA polymerase II transcription within the rRNA coding region and accumulation of R-loops, consistent with transcriptional interference between RNA polymerases I and II. Both olivetol and hypotonic stress also produced shared membrane-associated phenotypes, including reduced membrane lipid order, calcium redistribution, and plasma membrane blebbing. Importantly, this non-genotoxic nucleolar response depended on cholesterol-sensitive plasma membrane organization.

## Introduction

Naturally occurring phenolic compounds have attracted increasing attention as multifunctional bioactive molecules with antioxidant, anti-inflammatory, and metabolic regulatory properties. Among them, olivetol (5-pentylresorcinol) is a lipophilic alkylresorcinol found in certain lichens and insects and serves as a biosynthetic precursor of tetrahydrocannabinol (THC) in Cannabis sativa [1,2]. Beyond its role in cannabinoid biosynthesis, olivetol has been reported to exhibit a broad range of biochemical activities.

Previous studies have shown that olivetol possesses antioxidant and radical-scavenging properties, inhibits several enzymes linked to oxidative and neurodegenerative processes, and can modulate cannabinoid receptor signaling [1,3]. More recent in vivo studies have further suggested beneficial effects of olivetol in metabolic disorders, including reduced hepatic lipid accumulation, improved glucose and cholesterol metabolism, and preservation of pancreatic β-cell function [4,5]. Together, these observations indicate that olivetol is a biologically active small molecule with potentially broad physiological effects.

At the same time, the cellular mechanism of olivetol action remains poorly understood. Based on its amphipathic and lipophilic structure, olivetol can be expected to partition into cellular membranes and alter their physicochemical properties. Such membrane-associated activity could affect not only the plasma membrane, but also membrane-bound intracellular compartments, including the endoplasmic reticulum, mitochondria, and nuclear envelope [6–10]. Perturbation of these compartments could, in turn, influence ion homeostasis, organelle integrity, intracellular stress signaling, and ultimately cell viability [6,11–13].

This question is particularly important because biologically active small molecules are routinely evaluated not only for cytotoxicity but also for genotoxic potential, as reflected in the ICH guideline S2(R1) on genotoxicity testing and data interpretation for pharmaceuticals intended for human use. Importantly, membrane-active compounds may elicit nuclear stress responses indirectly: perturbation of the plasma membrane, endoplasmic reticulum, or mitochondrial membranes can disrupt calcium homeostasis, redox balance, and organelle integrity, thereby engaging DNA damage signaling without direct interaction with genomic DNA [11–14]. Whether olivetol perturbs membrane homeostasis in this way and whether such effects can propagate to nuclear stress responses remains unknown.

Here, we set out to define the cellular consequences of olivetol exposure in human cells, with particular emphasis on membrane-associated stress responses and genotoxicity. Unexpectedly, we found that olivetol does not induce detectable genomic DNA breaks, but instead activates a nucleolus-specific DDR at rDNA. This response closely phenocopies hypotonic stress, depends on Treacle and TOPBP1, and is associated with transcriptional interference and R-loop accumulation within the rDNA coding region. In parallel, olivetol and hypotonic stress induce closely related membrane-associated phenotypes, and the resulting nucleolar DDR depends on cholesterol-sensitive plasma membrane organization, pointing to a previously unrecognized membrane-to-nucleolus stress signaling pathway.

## Methods

### Cell culture and drug treatment

Human HeLa (ATCC® CCL-2™), HEK293 (ATCC® CRL-1573™), HAP1 and HCT116 (kindly provided by Dr. Nariman R Battulin, Institute of Cytology and Genetics SD RAS, Novosibirsk, Russia) cells were cultured in DMEM (PanEco) supplemented with 10% fetal bovine serum (FBS; HyClone/GE Healthcare) and penicillin/streptomycin. Human MV-4–11 (ATCC®CRL-9591™), THP1 (ATCC®TIB-202™), KG1 (ATCC®CRL-8031) and Kasumi-1 (ATCC®CRL-2724™) were cultured in RPMI (PanEco) supplemented with 10% fetal bovine serum (FBS; HyClone/GE Healthcare) and penicillin/streptomycin. The cells were cultured at 37°C in a conventional humidified CO2 incubator.

Cells were treated with 50–500 µM olivetol (Sigma-Aldrich, #152633) for the specified amount of time. DNA damage was induced by the treatment of cells with 20–70 µM etoposide (Sigma-Aldrich, #E1383) for 30 min. Hypotonic stress was applied by incubation the cells in 50% DMEM/50% H2O for 30 min. For channel inhibition, cells were pretreated with 50 µM 2-APB (Fluka, #42810), 10 µM DCPIB (Tocris, #1540), or 2 µM SEA0400 (Tocris, #6164) for 15 min, followed by olivetol treatment. For transcription inhibition experiments, cells were treated with 0.05 µg/ml actinomycin D (Biotium) for 3 h, 10 µM CX-5461 (SelleckChem) for 3 h, 50 µM DRB (Santa Cruz Biotechnology) for 3 h, or 1 µM Triptolide (Sigma-Aldrich) for 3 h.

For induction of ER stress, cells were treated with MG132 either at 50 µM for up to 3 h or at 500 nM for 72 h, with tunicamycin at 20 µg/mL for 72 h, or with CCT020312 at 10 µM for 3 h or 1 µM for 24 h. To induce cytoplasmic stress granule formation, cells were subjected to acute heat shock at 45°C for 30 min. To induce calcium influx and membrane blebbing, cells were treated with ionomycin at 1 µM for 60 min.

For membrane perturbation experiments, cells were pretreated with 1% Tween-20 in complete culture medium for 10 min and then exposed to olivetol or hypotonic stress without Tween-20 removal; with digitonin at 5 µg/mL in complete culture medium for 10 min and then exposed to olivetol or hypotonic stress without digitonin removal; or with 10 mM methyl-β-cyclodextrin (MβCD) in complete culture medium for 1 h.

### Gene knockdown

For CRISPR/Cas9-mediated knockout, two single guide RNAs (sgRNA) flanking a region of the target gene (TCOF1 or TOPBP1) were designed using the guide RNA design tool (www.atum.bio/eCommerce/cas9/input). The sgRNA targeting sequences were separately cloned into the sgRNA/Cas9 expression vector pSpCas9n(BB)-2A-Puro (PX462) V2.0 (Addgene #62987). A list of all oligonucleotides is provided in Supplementary Table S1. The plasmids were transfected into HeLa cells with LTX transfection reagent (Invitrogen). The transfectants were selected with 10 µg/ml puromycin for 24 h. After 24 h of puromycin selection, cells were switched to their normal culture medium. Clones of HeLa cells were obtained by limiting dilution into 96-well plates. Western blotting and indirect immunofluorescence was used to identify clones with TCOF1 or TOPBP1 depletion.

### Whole-cell extracts preparation and immunoblotting

Cells were lysed by incubation in RIPA buffer (150 mM NaCl, 1% Triton X-100, 0.5% sodium deoxycholate, 0.1% SDS, 50 mM Tris – HCl (pH 8.0) supplemented with Protease Inhibitor Cocktail (Bimake) and Phosphatase Inhibitor Cocktail (Bimake) for 30 min on ice. Next, the cell extracts were sonicated with a VirSonic 100 ultrasonic cell disrupter and stored at −70°C. The protein concentration was measured by the Bradford assay. Aliquots of each sample were separated by sodium dodecyl sulfate-polyacrylamide gelelectrophoresis and blotted onto polyvinylidene difluoride (PVDF) membranes (Amersham/GE Healthcare). The membranes were

blocked for 1 h in 2% ECL Advance blocking reagent (GE Healthcare) or 2% bovine serum albumin (BSA) (Sigma) in PBS containing 0.1% Tween 20 (PBS-T) followed by incubation overnight at 4°C with a primary antibody diluted in PBS-T containing 2% blocking reagent or 2% BSA. After three washes with PBS-T, the membranes were incubated for 1 h with the secondary antibodies (horseradish peroxidase-conjugated anti-rabbit or anti-mouse IgG) in PBS-T containing 2% blocking agent or 2% BSA. The immunoblots were visualized using a Pierce ECL plus western blotting substrate.

### Cell viability assay

Cells were seeded in 96-well plates at a density of 1500 cells per well in 100 μl media overnight. The adhered cells were treated with olivetol as indicated for 4–5 days. The media was replaced with fresh media with or without inhibitors every two days. Cell viability assays were performed using alamarBlue (ThermoFisher Scientific) according to the manufacturer’s instruction. A dose-response curve was used to assess drug response.

### Live-cell imaging and membrane visualization

For live-cell imaging and membrane labeling, cells were incubated with 100 µg/ml Concanavalin A – Alexa Fluor 488 conjugate (Biotium, #29016) and 1 µg/ml Hoechst 33,342 (Cell Signaling Technology) in HBSS for 30 min at 37°C.

For intracellular calcium analysis, the Fluo-4 Calcium Imaging Kit (Thermo Fisher Scientific, F10489) was used according to the manufacturer’s instructions. Cells were incubated with the Fluo-4 loading solution for 30 min at 30°C.

For visualization of plasma membrane blebbing and cell death, cells were incubated with 1 µg/ml Propidium Iodide (Fluka, #81845), 1 µg/ml Hoechst 33,342 (Cell Signaling Technology) and 1 µg/ml Calcein AM (Sigma, #56496) in complete culture medium for 30 min at 37°C.

After incubation, cells were washed and the medium was replaced with phenol red – free complete culture medium prior to live-cell imaging using a STELLARIS 5 confocal microscope (Leica Microsystems) equipped with an incubation chamber maintaining a humidified atmosphere at 37°C with 5% CO_2_ (objectives: HC PL APO × 63/1.40 oil CS2).

Images were typically acquired at 1024 × 1024 pixels with a scan speed of 200 ms per frame, capturing one frame per minute for 30–60 min. Image processing and montage assembly were performed using ImageJ (NIH).

### Fluorescence microscopy

For immunostaining, cells were grown on glass coverslips. All samples were fixed in CSK buffer (10 mM PIPES, pH 7.0, 100 mM NaCl, 1.5 mM MgCl_2_, 300 mM sucrose) supplemented with 1% paraformaldehyde (PFA) and 2.5% Triton X-100 for 15 min at room temperature. Cells were washed in PBS and then incubated with antibodies in PBS supplemented with 1% BSA and 0.05% Tween 20 for overnight at 4°C. Then the cells were washed with PBS three times (5 min each time). The primary antibodies bound to antigens were visualized using Alexa Fluor 488-conjugated, Alexa Fluor 594-conjugated or Alexa Fluor 647-conjugated secondary antibodies. The The list of antibodies used in this study is provided in Supplementary Table S2. DNA was counterstained with the fluorescent dye 4,6-diamino-2-phenylindole (DAPI) for 10 min at room temperature. The samples were mounted using Dako fluorescent mounting medium (Life Technologies). The immunostained samples were analyzed using a Zeiss AxioScope A.1 fluorescence microscope (objectives: Zeiss N-Achroplan 40 ×/0.65 and EC Plan-Neofluar 100 ×/1.3 oil; camera: Zeiss AxioCam MRm; acquisition software: Zeiss AxioVision Rel. 4.8.2; Jena, Germany) or STELLARIS 5 Leica confocal microscope (objectives: HC PL APO 63x/1.40 oil CS2). The images were processed using ImageJ software (version 1.44) and analyzed using CellProfiler software (version 3.1.5).

### Chromatin immunoprecipitation (ChIP) and ChIP-seq analysis

Living cells were fixed for 15 min with 1% formaldehyde at room temperature, and crosslinking was quenched by adding 125 mM glycine for 5 min. Cells were harvested in PBS, and nuclei were prepared by incubation in FL buffer (5 mM PIPES, pH 8.0, 85 mM KCl, 0.5% NP40) supplemented with Protease Inhibitor Cocktail (Bimake) and Phosphatase Inhibitor Cocktail (Bimake) for 30 min on ice. Next, chromatin was sonicated in RIPA buffer (10 mM Tris – HCl, pH 8.0, 140 mM NaCl, 1% Triton X-100, 0.1% sodium deoxycholate, 0.1% SDS) with a VirSonic 100 to an average length of 200–500 bp. Per ChIP reaction, 10–20 µg chromatin was incubated with 2–4 µg antibodies overnight at 4°C. The next day, Protein A/G Magnetic Beads (Thermo Scientific) were added to each sample and incubated for 4 h at 4°C. Immobilized complexes were washed two times for 10 min at 4°C in low salt buffer (20 mM Tris-HCl, pH 8.0, 150 mM NaCl, 2 mM EDTA, 0.1% SDS, 1% Triton X-100) and high salt buffer (20 mM Tris – HCl, pH 8.0, 500 mM NaCl, 2 mM EDTA, 0.1% SDS, 1% Triton X-100). Samples were incubated with RNase A (Thermo Scientific) for 30 min at room temperature. The DNA was eluted from the beads and de-crosslinked by proteinase K digestion for 4 h at 55°C and subsequent incubation at 65°C for 12 h. Next, DNA was purified using phenol/chloroform extraction and analyzed by qPCR. The qPCR primers used for ChIP analysis are listed in Supplementary Table S3. The sequencing libraries were then prepared with NEBNext Ultra II kit according manufacturer’s protocol. Final libraries were PCR amplificated and adapter dimers were cleaned with 1:1 MagPure magnetic beads (Magen Biotechnology). Resulted DNA was resuspended in 30 mkl 10 mM Tris-HCl buffer pH 8.0 and were sequenced on Illumina machine. Chip-seq reads were mapped to the reference human genome hg38 assembly using Bowtie v2.2.3 with the ‘–very-sensitive’ mode. Non-uniquely mapped reads, possible PCR and optical duplicates were filtered out using SAMtools v1.5. The bigWig files with the ratio of RPKM normalized ChIP-seq signal to the input were generated using deepTools v3.4.2 bamCompare function.

### RNA sequencing (RNA-seq)

HeLa cells were seeded in 12-well plates at appropriate numbers to allow cells to grow to ~90% confluence at the endpoint. For inhibitor treatment, cells were seeded 24 h before being treated with vehicle control (0.1% DMSO) or 300 μM olivetol for 30 min. Total RNA was isolated and purified by using TRIzol reagent (Thermo Fisher Scientific, 15,596,026) according to the manufacturer’s instructions. cDNA libraries for sequencing were prepared using NEBNext® rRNA Depletion Kit v2 (Human/mouse) (New England Biolabs, E7400, E7405) according to the manufacturer’s protocol. Next generation sequencing (NGS) was performed on the Illumina NextSeq targeting 50 millions of reads per sample.

RNA-seq reads were mapped to the reference human genome hg38 assembly using STAR 2.7.10a_alpha_220314 with following parameters: –outFilterType BySJout – outFilterMmkltimapNmax 20 –alignSJoverhangMin 8 –alignSJDBoverhangMin 1 –outFilterMismatchNmax 999 –outFilterMismatchNoverReadLmax 0.04 –alignIntronMin 20 – alignIntronMax 1,000,000 –alignMatesGapMax 1,000,000. Resulted SAM files were then sorted and converted to BAM format using samtools v 1.3.1.

For differential expression analysis read counts for transcripts were obtaining using featureCounts and NCBI RefSeq gtf file (downloaded from UCSC) with following parameters: -F GTF -M -s 0 -p -B -C -T 25 –countReadPair. Resulted raw count files were subjected to R package DESeq2.

### Neutral comet assay

Cell suspension at a concentration of 1 × 10^5^ cells/ml was mixed in a 1:1 ratio with Trevigen LMAgarose (#4250–050-02) at 37°C. The mixture was pipetted onto comet slides (Trevigen) pre-coated with a 1% normal melting point agarose (Sigma-Aldrich) base layer. The drop containing the cells was covered with a glass cover slip and incubated at 4°C for 5 min. After incubation, the coverslips were removed, and the slides were immersed in lysis solution (30 mM EDTA, 0.5% SDS, and 10 mM Tris – HCl, pH 8.0, 500 µg/ml proteinase K) and incubated at 37°C for 1 h. After lysis, the slides were washed three times for 5 min in PBS and incubated in 1×TBE (Tris-Borate-EDTA buffer) for 20 min at 4°C. Electrophoresis was performed in Trevigen electrophoresis system (#4250–050-ES) for 10 min at 4°C and 1 V/cm in 1×TBE. The comets were counterstained with SYBR Green for 1 h (1:3000; Thermo Scientific, #S7563) and then visualized using an inverted Nikon Eclipse Ti-E fluorescence microscope with a Nikon Intensilight C-HGFI light source (objective: Nikon Plan Fluor 4/0.13; camera: DS-Qi2). The images of the comets were analyzed using CellProfiler software (version 2.1.1 rev 6c2d896).

### Replication and transcription labelling

For 5-ethynyl-2’-deoxyuridine (EdU) incorporation, cells were incubated with 10 µM EdU (Life Technologies) in the presence of olivetol for 30 minutes at 37°C.Then, the cells were washed three times with PBS and fixed in CSK buffer (10 mM PIPES, pH 7.0, 100 mM NaCl, 1.5 mM MgCl2, 300 mM sucrose) supplemented with 1% paraformaldehyde (PFA) and 2.5% Triton X-100 for 15 min at room temperature. The samples were then processed using a Click-iT EdU Imaging Kit (Life Technologies) according to the manufacturer’s recommendations.

For EU incorporation, cells were incubated with olivetol for 3 hours, and during the final hour of incubation, 100 μM 5-ethynyl uridine (EU; Sigma-Aldrich) was added to the medium and cells were further incubated at 37°C. Then, the cells were washed three times with PBS and fixed in CSK buffer (10 mM PIPES, pH 7.0, 100 mM NaCl, 1.5 mM MgCl_2_, 300 mM sucrose) supplemented with 1% paraformaldehyde (PFA) and 2.5% Triton X-100 for 15 min at room temperature. The samples were then processed using a Click-iT EU Imaging Kit (Life Technologies) according to the manufacturer’s recommendations.

### Electron microscopy

Cells were fixed with 2.5% neutralized glutaraldehyde in the requisite buffer for 2 h at room temperature, post-fixed with 1% aqueous OsO4, and embedded in Epon. Sections of 100 nm thickness were cut and counterstained with uranyl acetate and lead citrate. Sections were examined and photographed with a JEM 1400 transmission electron microscope (JEOL, Japan) equipped with a QUEMESA bottom-mounted CCD-camera (Olympus SIS, Japan) and operated at 100 kV.

### Cell sorting

Cells were transiently transfected with the pICE-RNaseHI-WT-NLS-mCherry plasmid (Addgene #60365) using the Lipofectamine LTX transfection reagent (Invitrogen). Sixteen hours after transfection, cells were treated with olivetol or subjected to hypotonic stress, followed by fixation and fluorescence-activated cell sorting (FACS). Cell sorting was performed on a Sony SH800 Cell Sorter equipped with a 561 nm laser for red fluorescence detection. Sorting gates were established based on negative controls, and a minimum of 2 × 10^6^ events was collected for subsequent ChIP-qPCR analysis.

### S9.6 chromatin immunoprecipitation (DRIP-ChIP)

Cells were fixed for 15 min with 1% formaldehyde at room temperature and crosslinking was quenched with 125 mM glycine for 5 min. Cells were harvested in PBS and nuclei were isolated by incubation in FL buffer (5 mM PIPES, pH 8.0, 85 mM KCl, 0.5% NP40 supplemented with Protease Inhibitor Cocktail (Bimake) and Phosphatase Inhibitor Cocktail (Bimake) for 30 min on ice. Shromatin was sonicated in RIPA buffer (10 mM Tris – HCl, pH 8.0, 140 mM NaCl, 1% Triton X-100, 0.1% Na-deoxycholate, 0.1% SDS) with a VirSonic 100 ultrasonic cell disrupter to an average length of 300–500 bp. Next, RNase A and NaCl were added to final concentration 50 µg/ml and 400 mM, respectively, and incubated for 3 h at 37°C. Per immunoprecipitation reaction, ~10–20 µg of prepared chromatin were incubated with 5 µg S9.6 antibody overnight at 4°C. On the next day, Protein A/GMagnetic Beads (Thermo Scientific) were added in each sample and incubated for 4 h at 4°C. Immobilized complexes were washed two times for 10 min at 4°C in low salt buffer (20 mM Tris – HCl, pH 8.0, 150 mM NaCl, 2 mM EDTA, 0.1% SDS, 1% Triton X-100) and high salt buffer (20 mM Tris – HCl, pH 8.0, 500 mM NaCl, 2 mM EDTA, 0.1% SDS, 1% Triton X-100). The DNA was eluted from the beads and decrosslinked by Proteinase K digestion for 4 h at 55°C and overnight at 65°C. Next, the DNA was purified using phenol/chloroform extraction and analyzed by qPCR. The qPCR primers used to analyze ChIP DNA are included in Supplementary Table S4.

### Chemical synthesis of olivetol analogues

- 1-Butyl-3,5-dimethoxybenzene (BB 0282782; di-CH_3_-olivetol):

**Figure:**
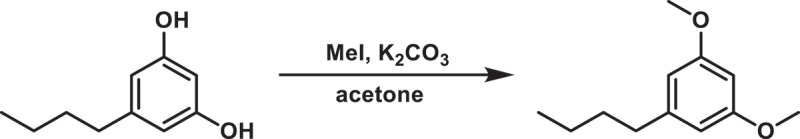

To stirred solution of substituted resorcinol (200 mg, 1.2 mmol, 1 eq.) in dry acetone (10 mL) was added potassium carbonate (500 mg, 3.6 mmol, 3 eq.) followed by addition of iodomethane (450 uL, 7.2 mmol, 6 eq.) at one portion. The reaction mixture was stirred at reflux for 2 days (TLC control). After cooling to room temperature, the suspension was filtered, solid was washed with acetone and filtrate was evaporated under reduced pressure. Crude product was purified by preparative column chromatography on silica gel using a 0% − 7% ethyl acetate gradient in hexane to provide title compound as a light-yellow oil. Yield 200 mg (85%).

The results of spectral analysis of the synthesized compounds are presented in Supplementary Table S5.

^1^H NMR (400 MHz, CDCl_3_) δ 6.35 (d, *J* = 2.3 Hz, 2 H), 6.30 (t, *J* = 2.3 Hz, 1 H), 3.78 (s, 6 H), 2.54 (t, *J* = 7.6 Hz, 2 H), 1.67–1.54 (m, 2 H), 1.32 (ddd, *J* = 5.4, 3.6, 1.8 Hz, 4 H), 0.89 (t, *J* = 7.0 Hz, 3 H).

LCMS [M+H^+^] 195.14

- 3-Butyl-5-methoxyphenol (BB 0282781; mono-CH_3_-olivetol):

**Figure:**
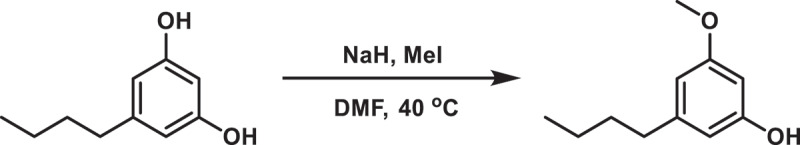

To stirred solution of substituted resorcinol (300 mg, 1.8 mmol, 1 eq.) in dry dimethylformamide was added sodium hydride dispersion in mineral oil (76 mg, 1.9 mmol, 1.05 eq.) at 0°C followed by addition of iodomethane (168 uL, 2.7 mmol, 1.5 eq.) by small portions. The reaction mixture was stirred at 40°C in sealed reaction vessel for 12 hours (TLC control). After cooling to room temperature, the solution was poured into cold water, extracted with ethyl acetate (2 × 30 mL), combined organic phases were washed with saturated sodium chloride solution (50 mL), dried over anhydrous sodium sulfate and concentrated under vacuum. Crude product was purified by preparative column chromatography on silica gel using a 0% − 9% ethyl acetate gradient in hexane to provide title compound as a light-yellow oil. Yield 190 mg (58%).

The results of spectral analysis of the synthesized compounds are presented in Supplementary Table S6.

^1^H NMR (400 MHz, CDCl_3_) δ 6.36–6.31 (m, 1 H), 6.29–6.26 (m, 1 H), 6.24 (t, *J* = 2.3 Hz, 1 H), 4.98 (s, 1 H), 3.77 (s, 3 H), 2.51 (t, *J* = 7.7 Hz, 2 H), 1.59 (p, *J* = 7.6 Hz, 2 H), 1.42–1.18 (m, 4 H), 0.89 (t, *J* = 6.9 Hz, 3 H).

LCMS [M+H^+^] 181.12

### Membrane tension estimation by tether-pulling experiments

Tether-pulling experiments were performed using a Bioscope Resolve atomic force microscope (AFM) (Bruker, Santa Barbara, CA, USA) mounted on an Axio Observer inverted fluorescence microscope (Carl Zeiss, Oberkochen, Germany). The system was equipped with a temperature-controlled stage, and all measurements were conducted at 37°C. MLCT-BIO-DC cantilevers (Bruker, Santa Barbara, CA, USA) with a nominal spring constant of 0.01 N/m were calibrated using the thermal tune method. Tether-pulling experiments were carried out as previously described [15]. Prior to measurements, cantilevers were cleaned with air plasma for 1 min (Plasma Cleaner, Harrick, NY, USA) and subsequently incubated with Concanavalin A CF®488A conjugate (Biotium) diluted in water to a final concentration of 1.0 mg/mL for 30 min at room temperature. Concanavalin A promotes strong binding of the coated AFM tip to carbohydrate moieties on the cell surface, enabling membrane tether formation during cantilever retraction. Measurements were performed in cell medium with approach/retraction velocity of 10 and 20 µm/s, a force setpoint of 500 pN, and a contact time of 1 s prior to retraction. Membrane tension (T_t) was estimated using the following equation:

$$T_{t}=\frac{1}{2\kappa }{\left(\frac{F}{2\pi }-\eta \frac{\nu }{c}\right)}^{2}$$

where κ is the bending modulus of the plasma membrane (assumed to be 2.7 × 10–19 N/m); F is the tether rupture force; ν is the tether pulling velocity; c is a correction factor with a value of 1.6; and η is the membrane viscosity estimated by comparing measurements obtained at two pulling velocities [15]. The tether rupture force was determined in a semi-automated manner using custom-written Python code incorporating a step-detection algorithm. For each condition and pulling velocity, at least 24 clearly resolved tether-pulling events were analyzed, collected from a minimum of 10 individual cells.

### Laurdan staining and GP analysis

To assess plasma membrane lipid order, cells were incubated with Laurdan at 5 µM in phenol red-free, serum-free DMEM for 20 min at 37°C. After incubation, cells were washed with the same medium to remove excess dye. Cells were then exposed to olivetol or hypotonic medium, and fluorescence images were acquired 10 min later. Laurdan fluorescence was recorded using two emission channels corresponding to the ordered and disordered membrane states: a green channel (430–460 nm) and a red channel (470–530 nm) after excitation with a 405 nm laser. Membrane lipid order was quantified by calculating the generalized polarization (GP) index according to the formula:

$$\mathrm{GP}=\frac{\mathrm{Igreen}-\mathrm{Ired}}{\mathrm{Igreen}+\mathrm{Ired}}$$

where Igreen and Ired are fluorescence intensities measured in the green and red channels, respectively.

### Statistical analysis

All quantitative data are presented as mean ± SD unless stated otherwise. Statistical significance was assessed using Student’s t-test, as indicated in the figure legends. Differences were considered statistically significant at *p* < 0.01.

## Results

### Olivetol triggers a nucleolar DNA damage response in the absence of detectable genomic DNA damage

**Figure 1.**
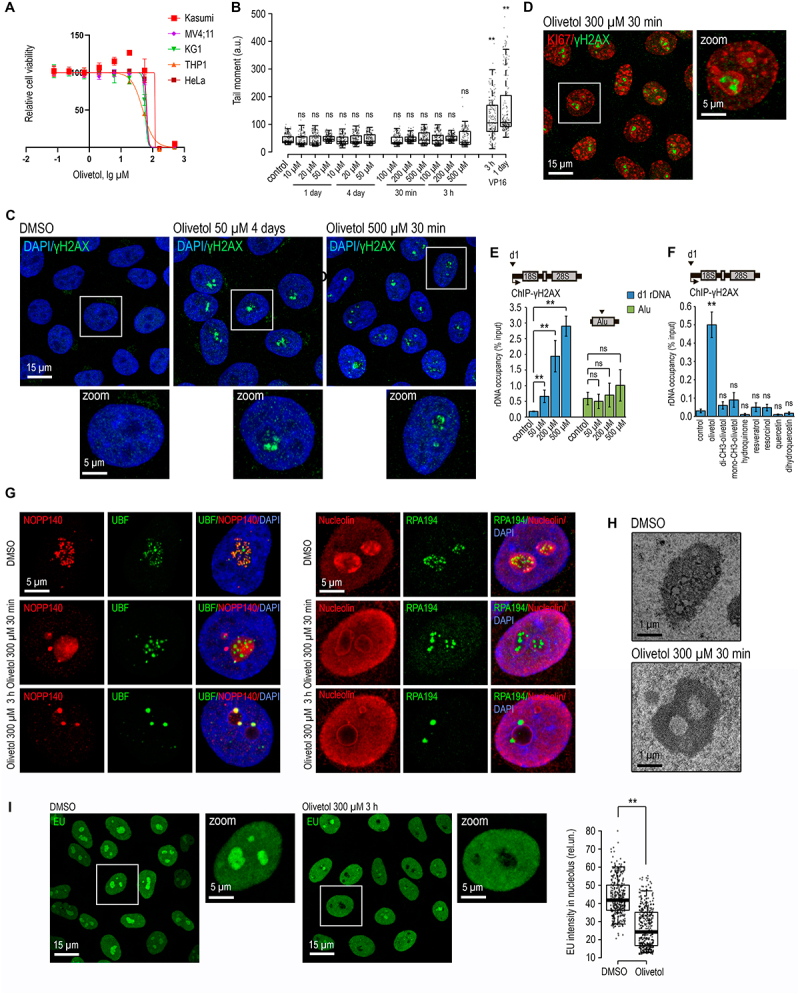
Olivetol induces a nucleolar DNA damage response (n-DDR) without detectable chromosomal DNA breaks. (A) Dose – response curves for Kasumi, MV4;11, KG1, THP1, and HeLa cells treated with increasing concentrations of olivetol for 4 days. Data are presented as mean ± SD (*n* = 3 biologically independent experiments). (B) HeLa cells were treated with 10–500 µM olivetol for the indicated time periods or with the DNA topoisomerase II inhibitor etoposide (VP16; 20 µM for 1 h). A neutral comet assay was performed; box plots show the tail moment. Horizontal lines represent the median. **, *p* < 0.01 by unpaired t-test; n.S., not significant (*n* > 500). (C) HeLa cells treated with DMSO (control) or with 50–500 µM olivetol for the indicated time periods were stained for γH2AX (green). DNA was stained with DAPI (blue). (D) HeLa cells were treated with 300 µM olivetol for 30 min and stained for γH2AX (green) and Ki67 (red). (E) HeLa cells were treated with 50–500 µM olivetol for 30 min. ChIP experiments were performed using antibodies against γH2AX. Enriched DNA was analyzed by qPCR with primer pair d1 specific for the rRNA gene promoter or ALU repeats, as indicated in the scheme. Data are presented relative to input. Values represent mean ± SD from at least three independent replicates. **, *p* < 0.01 by unpaired t-test; n.S., not significant. (F) HeLa cells were treated for 30 min with 300 µM olivetol, 300 µM di-CH3-olivetol (1-butyl-3,5-dimethoxybenzene; BB0282782), 300 µM mono-CH3-olivetol (3-butyl-5-methoxyphenol; BB0282781), 500 µM resveratrol, 500 µM resorcinol, 500 µM quercetin, or 500 µM dihydroquercetin. ChIP experiments were performed using antibodies against γH2AX. Enriched DNA was analyzed by qPCR with primer pair d1 specific for the rRNA gene promoter, as indicated in the scheme. Data are presented relative to input. Values represent mean ± SD from at least three independent replicates. (G) HeLa cells were treated with 300 µM olivetol for 30 min to 3 h and stained for UBF or RPA194 (green) together with NOPP140 or nucleolin (red). DNA was stained with DAPI (blue). (H) HeLa cells were treated with 300 µM olivetol for 30 min and analyzed by transmission electron microscopy (TEM). (I) HeLa cells treated with DMSO (control) or with 300 µM olivetol for 3 h were labeled with 5-ethynyl uridine (EU). EU incorporation was detected by click chemistry. The graph on the right shows quantification of nucleolar EU fluorescence intensity in more than 200 cells. **, *p* < 0.01 by unpaired t-test.

To characterize the cellular effects of olivetol, its impact on cell viability was first assessed. Several human cell lines of different origins, including HeLa, KG1, MV-4–11, THP1, and Kasumi-1, were exposed to a wide range of olivetol concentrations for 90–120 hours. The calculated IC_5__0_ values were comparable across all tested lines, ranging from 50 to 100 µM (57–65 µM for HeLa; 56–58 µM for KG1; 63–86 µM for MV-4–11; 49–58 µM for THP1; and 97–118 µM for Kasumi-1), indicating a moderate cytotoxic potential of the compound (Figures 1(A) and S1(A)).

To determine whether olivetol induces genotoxic stress, we assessed DNA damage in treated cells. HeLa cells were treated with olivetol at sub-cytotoxic doses (≤IC50; 50 µM for up to 4 days) or short-term high doses (≥IC50; 100–500 µM for 0.5–3 h), and the level of DNA damage was assessed using neutral comet assay and immunofluorescence staining of phosphorylated histone H2AX at Ser139 (γH2AX). As a positive control, cells were treated with etoposide, a topoisomerase II poison known to induce persistent DNA double-strand breaks. The comet assay revealed no increase in DNA strand breaks under any treatment condition (Figure 1(B)). However, immunostaining for γH2AX revealed a striking pattern: after 30 minutes of exposure to high concentrations (100–500 µM) or after several days at 50 µM, discrete intranuclear γH2AX foci appeared (Figure 1(C)). Strikingly, these γH2AX foci were confined to nucleoli, as confirmed by co-staining of γH2AX and the nucleolar marker Ki-67 (Figure 1(D)). Furthermore, EdU pulse-labeling demonstrated that this nucleolar DNA damage – like response (n-DDR) occurs independently of DNA replication and was equally observed in both replicating (S-phase) and non-replicating cells (Figure S1(B)).

The nucleolus is the site of ribosomal RNA (rRNA) synthesis. To verify whether γH2AX accumulation occurred at rDNA loci, chromatin immunoprecipitation (ChIP) was performed with antibodies against γH2AX, followed by qPCR for rDNA promoter sequences. As expected, γH2AX enrichment was strongly increased at the rDNA promoter region in olivetol-treated cells (Figure 1(E)). In contrast, no increase was detected at ALU repeats, used as a non-rDNA control (Figure 1(E)), indicating locus-specific activation of DDR within the rDNA array. Importantly, this response was observed not only in HeLa cells but also in HEK293, HAP1, and HCT116 cells (Figure S1(C)), demonstrating that this phenomenon is not cell line – specific. Together, these results demonstrate that olivetol activates a nucleolus-specific DDR (n-DDR) rather than a global, genome-wide DNA damage response.

To test whether this effect is unique to olivetol, we next examined structurally related resorcinol derivatives, including mono- and di-methylated olivetol analogs (mono-CH_3_-olivetol and di-CH_3_-olivetol), as well as resveratrol, resorcinol, and hydroquinone. HeLa cells were incubated with each compound (100–500 µM, 1–24 h), and the γH2AX enrichment at rDNA promoters was assessed by ChIP-qPCR. Remarkably, none of the tested analogs or related phenolic compounds reproduced the olivetol-induced n-DDR, even at the highest concentrations (Figure 1(F)), suggesting that this effect is structurally specific to the unmodified olivetol molecule.

It is known that activation of n-DDR by genotoxic agents targeting rDNA typically leads to disruption of nucleolar architecture [16–18]. The nucleolus consists of three concentric sub-compartments: the fibrillar centers (FC), the dense fibrillar component (DFC), and the granular component (GC), which contains assembling ribosomal subunits [19]. To assess how olivetol affects nucleolar structure, immunostaining was performed for representative markers of these compartments: RPA194 and UBF (FC), NOPP140 (DFC), and nucleolin (NCL) (GC). In control cells, these markers exhibited the characteristic tripartite organization of the nucleolus, with FCs surrounded by DFC and GC layers (Figure 1(G)). Following olivetol treatment, progressive nucleolar disorganization was observed. Within 30 minutes, NCL and NOPP140 redistributed from the nucleolus to the nucleoplasm, and after 1–3 hours, nucleolar ‘caps’ formed, consistent with FC fusion under conditions of transcriptional repression (Figure 1(G)). Electron microscopy further confirmed nucleolar structural collapse after 30 minutes of exposure (Figures 1(H) and S1(D)).

Because nucleolar disintegration typically accompanies transcriptional silencing of rDNA [20], we next measured nascent RNA synthesis using 5-ethynyl uridine (EU) incorporation. Olivetol treatment resulted in complete loss of nucleolar EU incorporation, indicating robust repression of rRNA transcription (Figure 1(I)).

Together, these findings demonstrate that olivetol triggers a nucleolar DNA damage – like response (n-DDR) accompanied by nucleolar disorganization and rRNA transcriptional arrest, despite the absence of detectable genomic DNA damage.

### Olivetol-induced n-DDR requires Treacle and TOPBP1

**Figure 2.**
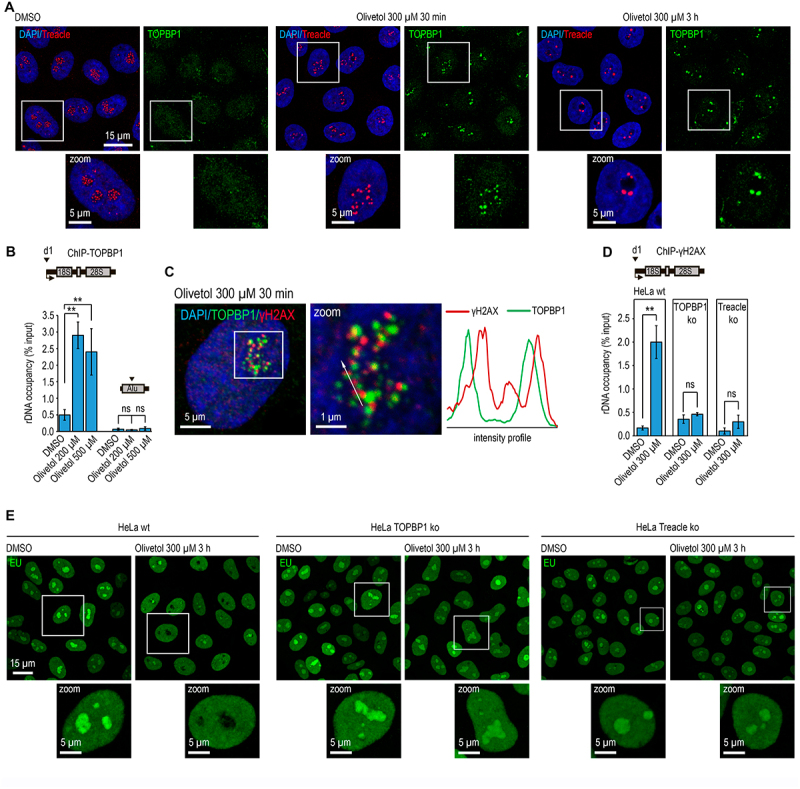
Olivetol induces a Treacle/TOPBP1-dependent n-DDR. (A) HeLa cells treated with DMSO (control) or with 300 µM olivetol for 30 min to 3 h were co-immunostained for TOPBP1 (green) and Treacle (red). DNA was stained with DAPI (blue). (B) HeLa cells were treated with 200–500 µM olivetol for 30 min. ChIP experiments were performed using antibodies against TOPBP1. Enriched DNA was analyzed by qPCR with primer pair d1 specific for the rRNA gene promoter or ALU repeats, as indicated in the scheme. Data are presented relative to input. Values represent mean ± SD from at least three independent replicates. **, *p* < 0.01 by unpaired t-test; n.S., not significant. (C) HeLa cells were treated with 300 µM olivetol for 30 min and co-immunostained for TOPBP1 (green) and γH2AX (red). DNA was stained with DAPI (blue). Colocalization analysis was performed on merged images. Graphs illustrate quantification of fluorescence distribution, in arbitrary units, along the lines shown in the images. (D) HeLa wild-type (HeLa wt) cells and HeLa cells with TOPBP1 knockout (TOPBP1 ko) or Treacle knockout (Treacle ko) were treated with DMSO (control) or 300 µM olivetol for 30 min. ChIP experiments were performed using antibodies against γH2AX. Enriched DNA was analyzed by qPCR with primer pair d1 specific for the rRNA gene promoter, as indicated in the scheme. Data are presented relative to input. Values represent mean ± sd from at least three independent replicates. **, *p* < 0.01 by unpaired t-test; n.S., not significant. (E) HeLa wt, TOPBP1 ko, and Treacle ko cells were treated with DMSO (control) or 300 µM olivetol for 3 h. Cells were then labeled with 5-ethynyl uridine (EU), and EU incorporation was detected by click chemistry.

Previous studies, including our own, have shown that nucleolar DNA damage responses depend on the interaction between the DNA damage scaffold protein TOPBP1 and the nucleolar protein Treacle, which organizes the fibrillar centers of the nucleolus [17,18,21,22]. To determine whether the same mechanism operates during olivetol-induced n-DDR, we examined the localization and interaction of these proteins in olivetol-treated cells. Immunostaining for TOPBP1 and Treacle revealed that olivetol treatment induced robust recruitment of TOPBP1 to nucleoli, where it co-localized with Treacle within the FC region (Figure 2(A)). Consistent with this observation, ChIP analysis showed a strong enrichment of TOPBP1 at the rDNA promoter upon olivetol treatment (Figure 2(B)). Furthermore, co-immunostaining for γH2AX and TOPBP1 revealed a tight spatial association between γH2AX-positive sites and TOPBP1-enriched FCs (Figure 2(C)), suggesting that nucleolar DNA damage signaling is coordinated at these sites.

To test whether TOPBP1 and Treacle are required for olivetol-induced n-DDR, we used HeLa knockout cell lines lacking either TOPBP1 (TOPBP1 ko) or Treacle (Treacle ko) (Figs. S2A and S2B). Cells were treated with olivetol, and γH2AX enrichment at rDNA promoters was measured by ChIP-qPCR. As expected, loss of either TOPBP1 or Treacle completely abolished olivetol-induced γH2AX accumulation at rDNA (Figure 2(D)), demonstrating that both proteins are required for olivetol-induced n-DDR.

As shown above, olivetol treatment suppresses rDNA transcription, a hallmark of nucleolar DDR. To determine whether this transcriptional silencing is directly mediated by n-DDR signaling, nascent RNA synthesis was monitored by 5-ethynyl uridine (EU) incorporation in TOPBP1 knockdown or Treacle knockout HeLa cells exposed to olivetol. The absence of either protein significantly prevented olivetol-induced inhibition of rRNA synthesis (Figure 2(E)), indicating that rDNA transcriptional repression occurs downstream of n-DDR activation.

To determine whether n-DDR contributes to olivetol cytotoxicity, cell viability and IC50 values were compared between wild-type, TOPBP1 ko, and Treacle ko HeLa cells. IC50 values were comparable between wild-type, TOPBP1 ko, and Treacle ko cells (Figure S2(C)).

Together, these results demonstrate that olivetol triggers a canonical Treacle – TOPBP1-dependent nucleolar DNA damage response leading to rDNA transcriptional repression. However, olivetol cytotoxicity occurs independently of n-DDR activation.

### Olivetol mimics hypotonic stress to trigger transcriptional conflict and R-loop – dependent nucleolar DDR

Only two conditions have previously been shown to induce nucleolar DDR without detectable genome-wide DNA damage: targeted rDNA cleavage by the homing endonuclease I-PpoI [17,20,23–25] and hypotonic stress [23]. Because I-PpoI represents an artificial system relying on expression of an exogenous nuclease, we focused on hypotonic stress as a physiological reference condition and asked whether olivetol-induced n-DDR follows a similar mechanism.

**Figure 3.**
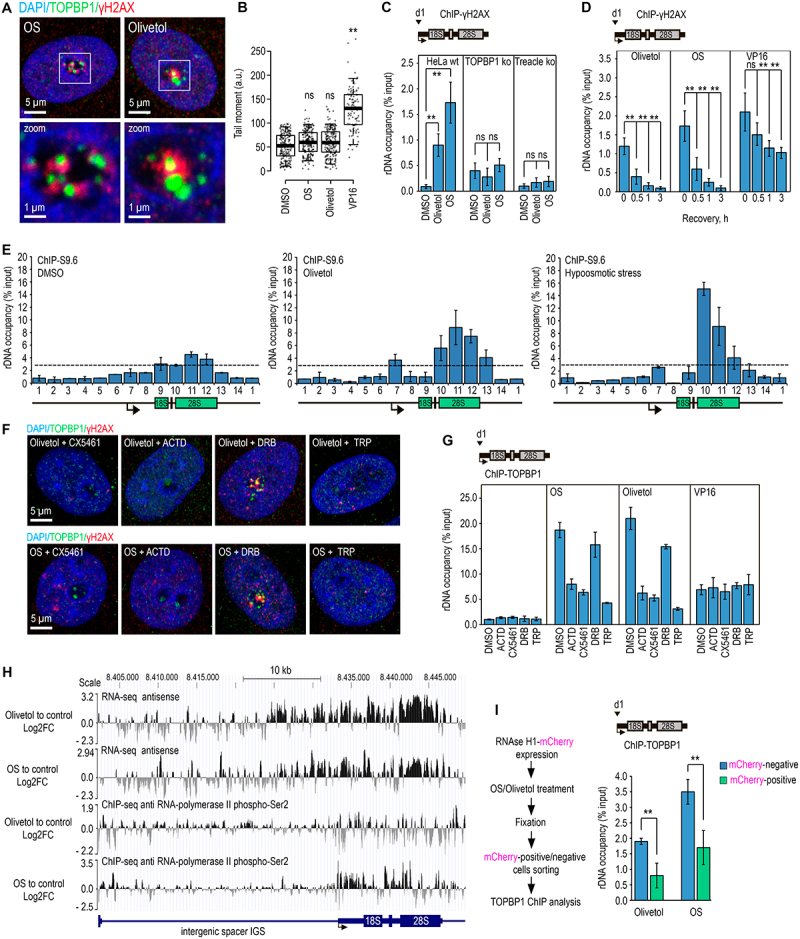
Olivetol and hypotonic stress promote R-loop formation and TOPBP1 recruitment via RNA polymerase I – driven sense and RNA polymerase II – driven antisense transcription at rDNA. (A) HeLa cells were treated with 300 µM olivetol or subjected to hypotonic stress (OS) for 30 min. Cells were co-immunostained for TOPBP1 (green) and γH2AX (red). DNA was stained with DAPI (blue). (B) HeLa cells were treated with DMSO, 300 µM olivetol, hypotonic stress (OS), or 20 µM VP16 for 30 min. A neutral comet assay was performed; box plots show the tail moment. Horizontal lines represent the median. **, *p* < 0.01 by unpaired t-test; n.S., not significant (*n* > 500). (C) HeLa wt, TOPBP1 ko, and Treacle ko cells were treated with DMSO (control), 300 µM olivetol, or hypotonic stress (OS) for 30 min. ChIP experiments were performed using antibodies against γH2AX. Enriched DNA was analyzed by qPCR with primer pair d1 specific for the rRNA gene promoter, as indicated in the scheme. Data are presented relative to input. Values represent mean ± sd from at least three independent replicates. **, *p* < 0.01 by unpaired t-test; n.S., not significant. (D) HeLa cells were treated with 300 µM olivetol, subjected to hypotonic stress (OS), or exposed to 70 µM VP16 for 30 min. Following treatment, cells were washed and incubated in fresh complete medium for 0.5, 1, or 3 h to allow recovery. Cells were then analyzed by ChIP-qPCR using antibodies against γH2AX and primer pair d1 specific for the rRNA gene promoter, as indicated in the scheme. Data are presented relative to input. Values represent mean ± sd from at least three independent replicates. **, *p* < 0.01 by unpaired t-test; n.S., not significant. (E) R-loop accumulation was measured by DRIP-ChIP using the S9.6 antibody in control cells (DMSO), cells treated with 300 µM olivetol for 30 min, or cells subjected to hypotonic stress (OS) for 30 min. DRIP-ChIP was followed by qPCR using the indicated rDNA amplicons shown below the graphs. Data are presented relative to input. Values represent mean ± sd from at least three independent experiments. (F) HeLa cells were treated for 3 h with Pol I inhibitors, actinomycin D (ACD; 0.05 µg/mL) or CX5461 (10 µM), or with Pol II inhibitors, 5,6-dichlorobenzimidazole 1-β-D-ribofuranoside (DRB; 50 µM) or triptolide (trp; 1 µM). During the last 0.5 h of treatment, cells were exposed to 300 µM olivetol or hypotonic stress. Cells were co-immunostained for TOPBP1 (green) and γH2AX (red). DNA was stained with DAPI (blue). (G) HeLa cells were treated as described in (F), except that during the last 0.5 h of treatment they were exposed to 300 µM olivetol, hypotonic stress, or 70 µM etoposide (VP16). ChIP experiments were performed using antibodies against TOPBP1. Enriched DNA was analyzed by qPCR with primer pair d1 specific for the rRNA gene promoter, as indicated in the scheme. Data are presented relative to input. Values represent mean ± sd from at least three independent replicates. (H) HeLa cells were treated with 300 µM olivetol or subjected to hypotonic stress (OS) for 30 min; DMSO-treated cells served as a control. Cells were then processed either for RNA-seq or for ChIP-seq using antibodies against RNA polymerase II phospho-Ser2. Shown are profiles of antisense transcription across the ribosomal DNA (rDNA) unit, represented as log10 Fold change in transcription levels for OS versus DMSO and olivetol versus DMSO. Distribution profiles of RNA polymerase II phospho-Ser2 ChIP-seq signal across the rDNA unit are also shown as log2 Fold change for olivetol versus DMSO and OS versus DMSO. (I) HeLa cells were transiently transfected with a plasmid encoding an RNase H – mCherry fusion protein. Sixteen hours after transfection, cells were treated with 300 µM olivetol or subjected to hypotonic stress (OS) for 30 min. Cells were then fixed and sorted into mCherry-positive and mCherry-negative populations. Each population was subjected separately to ChIP-qPCR using antibodies against TOPBP1 and primer pair d1 specific for the rRNA gene promoter, as indicated in the scheme. Data are presented relative to input. Values represent mean ± sd from at least three independent replicates. **, *p* < 0.01 by unpaired t-test; n.S., not significant.

Indeed, the n-DDR induced by olivetol closely resembled the response induced by hypotonic stress. Both stimuli induced γH2AX phosphorylation specifically within nucleoli (Figures 3(A) and S3), with no detectable DNA damage elsewhere in the genome, as confirmed by neutral comet assay (Figure 3(B)). In both cases, γH2AX accumulation in rDNA was dependent on Treacle and TOPBP1 (Figure 3(C)), consistent with a canonical nucleolar DDR mechanism. Furthermore, both stresses exhibited similar recovery kinetics: γH2AX levels at rDNA promoters returned to baseline within ~1 hour after stress removal, in contrast to persistent DDR signaling observed after genotoxic damage (e.g., through etoposide-induced DSBs) (Figure 3(D)).

We have previously shown that hypotonic stress activates nucleolar DNA damage response through stabilization of R-loops within rDNA [23]. We therefore asked whether olivetol induces a similar R-loop – dependent mechanism. To assess this, the distribution of R-loops was analyzed across rDNA using DRIP-ChIP (DNA:RNA immunoprecipitation followed by qPCR) with S9.6 antibodies in control HeLa cells and in cells exposed to olivetol or hypotonic stress. Both treatments caused a significant accumulation of R-loops within the rRNA coding region but not within the intergenic spacer (IGS) (Figure 3(E)). The strongest accumulation occurred within the region encoding 28S rRNA (Figure 3(E)).

Because R-loops in the rRNA coding region are typically associated with Pol I activity, we next examined whether the activation of n-DDR depends on Pol I-mediated transcription. Cells were treated with olivetol or hypotonic medium in the presence of Pol I inhibitors (low-dose actinomycin D or CX5461) and inhibitors of Pol II, including the elongation inhibitor DRB and the initiation inhibitor triptolide (TRP). As expected, both actinomycin D and CX5461 abolished n-DDR activation (as measured by γH2AX and TOPBP1 accumulation in nucleoli and ChIP-qPCR analysis) under both olivetol and hypotonic conditions (Figure 3(F,G)). Unexpectedly, inhibition of Pol II initiation by triptolide (TRP), but not elongation inhibition by DRB, also prevented n-DDR activation. These results indicate that Pol I transcription alone is insufficient to trigger n-DDR and that Pol II – dependent transcription is also required. Importantly, n-DDR triggered by etoposide was insensitive to both Pol I and Pol II inhibitors (Figure 3(G)), indicating that the nucleolar response to olivetol and hypotonic stress represents a highly specific transcription-dependent mechanism that is distinct from canonical DDR.

Pol II transcription within rDNA occurs in the antisense direction relative to Pol I – driven rRNA transcription. A prominent example of such antisense transcription is the long non-coding RNA PAPAS [26–28], which is transcribed across the rRNA coding region by Pol II and is known to be upregulated under hypotonic stress [29]. To determine whether olivetol induces a similar antisense transcriptional program, we analyzed RNA-seq data and ChIP-seq profiles using antibodies against the elongating Ser2-phosphorylated form of Pol II (Pol II-pSer2). Both olivetol and hypotonic stress strongly increased antisense transcription across the rDNA coding region, extending from the terminator toward the promoter, and this correlated with increased Pol II-pSer2 occupancy (Figure 3(H)). Although minor antisense peaks were also observed in the IGS region, these were not further analyzed, as R-loop enrichment was not detected there by DRIP-ChIP. Taken together, these results suggest that both olivetol and hypotonic stress induce bidirectional transcription within rDNA, in which Pol I and Pol II move in opposite directions, likely leading to transcriptional collisions between Pol I and Pol II and stabilization of R-loops, which in turn trigger n-DDR activation.

Finally, to directly test whether R-loop stabilization is required for n-DDR, we expressed mCherry-tagged RNase H1 in HeLa cells. Twenty-four hours after transfection, cells were exposed to olivetol or hypotonic stress, and mCherry-positive and mCherry-negative populations were separated by flow cytometry. Subsequent ChIP-qPCR analysis with anti-TOPBP1 antibodies revealed that RNase H1 expression significantly reduced TOPBP1 occupancy at rDNA promoters under both treatments, although not completely (Figure 3(I)). This partial reduction suggests that R-loops represent an important, but not the sole, upstream signal leading to n-DDR activation.

In summary, our data indicate that olivetol and hypotonic stress activate nucleolar DDR through a shared mechanism involving transcriptional conflict between Pol I and Pol II and stabilization of R-loops within rDNA, which facilitates TOPBP1 recruitment and γH2AX phosphorylation. While R-loop formation plays a central role in this process, additional stress-related factors, including ER stress and chromatin reorganization, may further contribute to nucleolar DDR activation. These findings established a shared downstream mechanism of olivetol- and hypotonic stress-induced n-DDR, but did not explain how these stimuli are initially sensed by the cell.

### Olivetol and hypotonic stress trigger a common membranotropic stress response

Because olivetol and hypotonic stress triggered nucleolar DDR through an indistinguishable Treacle – TOPBP1 pathway, we next asked whether these stimuli also induce common upstream cellular responses. A defining feature of hypotonic stress is osmotic swelling caused by water influx. We therefore first tested whether olivetol induces a similar morphological response. Unlike hypotonic treatment, however, olivetol did not cause a detectable increase in cell volume or visible chromatin relaxation (Figure S4(A)), indicating that although olivetol phenocopies hypotonic stress at the level of nucleolar DDR, it does not reproduce its osmotic component.

**Figure 4.**
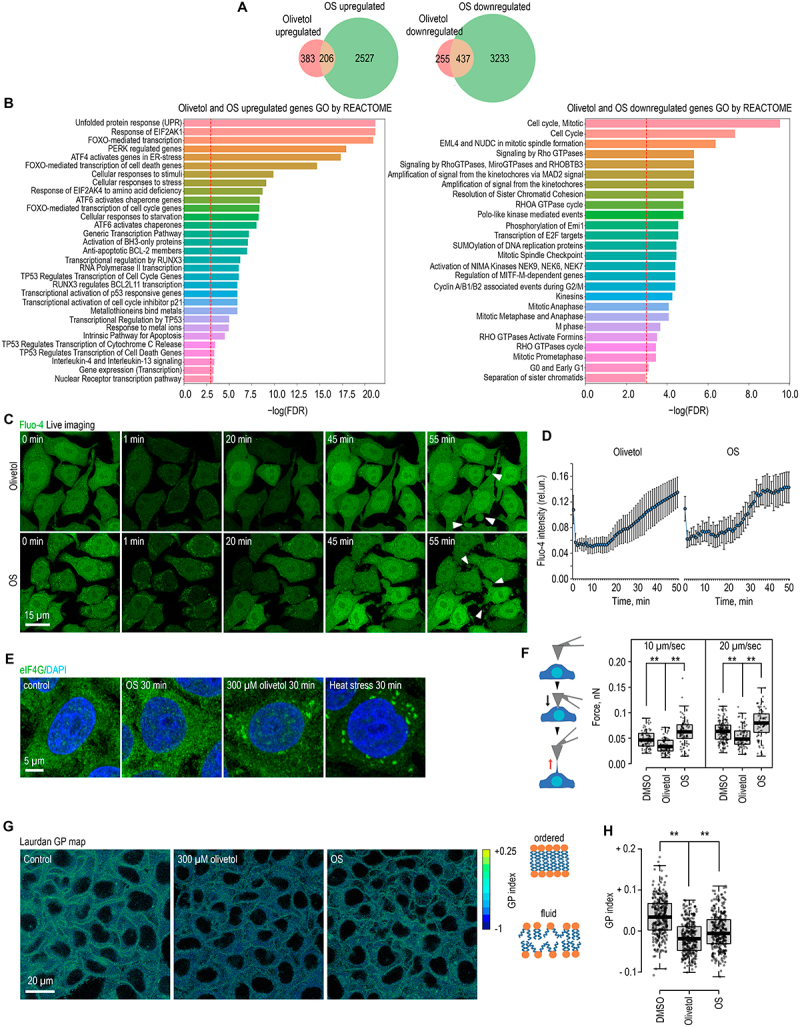
Olivetol and hypotonic stress induce similar cellular responses and alter plasma membrane mechanics. (A) HeLa cells were treated with 300 µM olivetol or subjected to hypotonic stress (OS) for 30 min. Overlaps of upregulated and downregulated genes between the olivetol- and hypotonic stress – treated RNA-seq datasets are shown. (B) HeLa cells were treated with 300 µM olivetol or subjected to hypotonic stress (OS) for 30 min. Gene Ontology (GO) enrichment analysis was performed for the overlapping upregulated and downregulated genes. Values are presented as −log10(FDR). (C) Calcium dynamics were visualized using the fluorescent probe Fluo-4. Cells were loaded with Fluo-4, washed, and then exposed to 300 µM olivetol or hypotonic stress (OS) for the indicated time periods. White arrowheads indicate forming membrane blebs. (D) Cells were treated as described in (C). Fluorescence intensity was quantified in each frame using CellProfiler across more than 50 individual cells. Data are presented as mean ± sd. Representative fluorescence intensity kinetics are shown in the plots on the right. (E) HeLa cells were treated with 300 µM olivetol for 30 min, subjected to osmotic stress (OS) for 30 min, or exposed to heat shock at 45°C for 30 min as a positive control for stress granule formation. Cells were then fixed and immunostained with antibodies against eIF4G (green). DNA was stained with DAPI (blue). (F) a schematic of the tether-pulling assay is shown to the left of the graph. Briefly, the cantilever was brought into contact with the plasma membrane and then retracted to pull a membrane tether; the force was measured at the moment of tether rupture. Cells were treated with 300 µM olivetol or subjected to osmotic stress (OS), and tether-pulling experiments were initiated immediately thereafter. Measurements were performed at retraction velocities of 10 and 20 µm/s, and at least 24 clearly resolved tether-pulling events from a minimum of 10 individual cells were analyzed for each condition and pulling velocity. See Methods for details. **, *p* < 0.01 by unpaired t-test. (G) Cells were stained with Laurdan and then treated with 300 µM olivetol or subjected to osmotic stress (OS); fluorescence images were acquired 10 min later. Laurdan fluorescence was recorded in two emission channels corresponding to ordered and disordered membrane states: a green channel (430–460 nm) and a red channel (470–530 nm) following excitation with a 405 nm laser. Membrane lipid order was quantified by calculating the generalized polarization (GP) index. Representative GP maps of the cells are shown. (H) Cells were analyzed as described in (G). The graph shows quantification of the GP index at the plasma membrane from more than 200 cells. **, *p* < 0.01 by unpaired t-test.

To compare cellular responses more broadly, we performed RNA-seq analysis of cells exposed to olivetol or hypotonic stress. Differential expression profiling revealed substantial overlap between the two conditions (Figure 4(A)). Downregulated genes were enriched for pathways related to cell cycle progression and mitotic control, whereas upregulated genes were enriched for EIF2A-dependent signaling, PERK, FOXO, ATF4/6 transcriptional programs, and the unfolded protein response (UPR), consistent with activation of ER stress pathways (Figure 4(B)) [30,31]. These data suggested that olivetol and hypotonic stress induce a similar ER stress-like transcriptional program.

To determine whether this program reflects classical proteotoxic ER stress, we examined stress granule formation by immunostaining for eIF4G. As expected, acute heat shock robustly induced eIF4G-positive cytoplasmic stress granules (Figures 4(E) and S4(B)). In contrast, hypotonic stress did not induce detectable stress granules, whereas olivetol caused only a modest increase, arguing against classical proteotoxic stress as a common upstream trigger [12,32].

Because disruption of calcium homeostasis is another hallmark of ER stress [12,33], intracellular calcium dynamics were monitored using Fluo-4. Both olivetol and hypotonic stress caused gradual depletion of Ca^2 +^ from the ER followed by cytosolic accumulation (Figure 4(C,D); video-1 and video-2). This calcium release was not suppressed by the IP3 receptor inhibitor 2-APB, indicating that it does not occur through canonical ER Ca^2 +^ channels, whereas inhibitors of plasma membrane ion transport affected only the later phase of cytosolic Ca^2 +^ elevation (Figure S4(C)). These data indicate that both stimuli induce a nonspecific redistribution of intracellular calcium, likely as a consequence of membrane perturbation.

Consistent with this interpretation, prolonged exposure to either olivetol or hypotonic stress led to pronounced plasma membrane blebbing (Figure 4(C) and S4D; video-1, video-2 and video-3). Concanavalin A staining confirmed extensive membrane deformation under both conditions (Figs. S4E and S4F; video-4, video-5). At sublethal doses, olivetol-induced blebbing was accompanied by decreased fluorescence of calcium indicators and increased propidium iodide uptake, indicating compromised plasma membrane integrity and reduced cell viability (Figure S4(G); video-6). Moreover, treatment of fixed HeLa cells with supraphysiological concentrations of olivetol (>1 mM) enabled entry of DAPI and antibodies even in the absence of detergent permeabilization, demonstrating that olivetol is intrinsically capable of permeabilizing cellular membranes (Figure S4(H)). Together, these observations indicate that both olivetol and hypotonic stress converge at the level of membrane perturbation, consistent with the amphiphilic structure of olivetol.

Recent studies have shown that ER stress can be triggered not only by accumulation of misfolded proteins, but also by disturbances in membrane lipid organization, a process referred to as lipid bilayer stress (LBS) [34–37]. Consistent with this model, genes upregulated under both olivetol and hypotonic conditions included not only canonical UPR targets such as CHAC1, DDIT3, ATF4, HERPUD1, and SESN2, but also regulators of lipid homeostasis and membrane remodeling, including NR1H3 (LXRα), ABCG1, TMEM86A, GPCPD1, and PLPP6. The coexistence of these signatures suggests that both stimuli induce a membrane-associated stress program more consistent with LBS than with classical proteotoxic stress.

To compare their effects on plasma membrane mechanics, we next performed AFM tether-pulling experiments. Surprisingly, olivetol and hypotonic stress had opposite effects: hypotonic stress increased membrane tension, whereas olivetol decreased it (Figure 4(F)). We therefore asked whether they might instead share a common effect on lipid organization. Laurdan imaging showed that generalized polarization (GP) values decreased modestly but significantly under both conditions (Figure 4(G)), indicating reduced membrane lipid order despite opposite effects on membrane tension [38–42].

Together, these results indicate that olivetol and hypotonic stress elicit a shared membrane-associated stress response characterized by reduced lipid order, calcium redistribution, membrane deformation, and induction of a transcriptional program consistent with ER and lipid bilayer stress. We therefore next asked which of these shared membrane-associated phenotypes, if any, are functionally required for n-DDR activation.

### Nucleolar DDR induced by olivetol and hypotonic stress depends on cholesterol-sensitive plasma membrane organization

Having established that both olivetol and hypotonic stress trigger a similar membranotropic stress response, we next asked whether the nucleolar DDR induced by these treatments is directly linked to any of the associated phenotypes described above, including ER stress, lipid bilayer stress, plasma membrane blebbing, or calcium redistribution.

**Figure 5.**
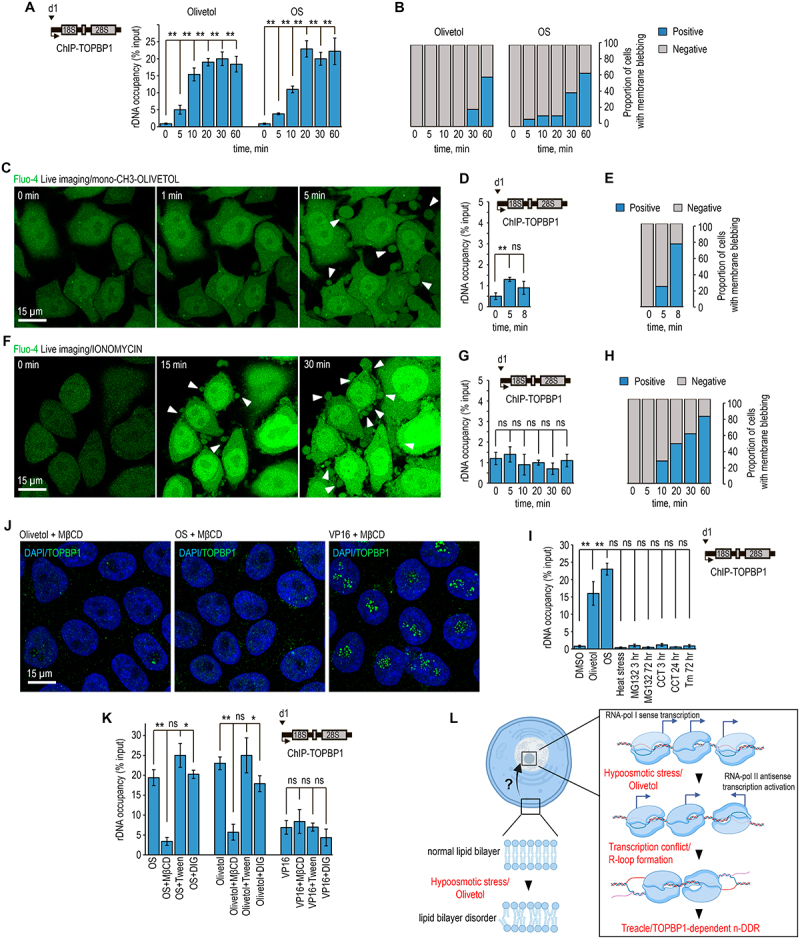
Plasma membrane perturbation promotes TOPBP1 recruitment to rDNA and activation of the nucleolar DNA damage response. (A) HeLa cells were treated with 300 µM olivetol or subjected to hypotonic stress for the indicated time periods. ChIP experiments were performed using antibodies against TOPBP1. Enriched DNA was analyzed by qPCR with primer pair d1 specific for the rRNA gene promoter, as indicated in the scheme. Data are presented relative to input. Values represent mean ± sd from at least three independent replicates. (B) Cells were treated as described in (a) and loaded with Fluo-4. At the indicated time points, cells were fixed, and the proportion of cells displaying pronounced membrane blebbing was quantified. (C) Calcium dynamics were visualized using the fluorescent probe Fluo-4. Cells were loaded with Fluo-4, washed, and then exposed to 300 µM mono-CH3-olivetol (3-butyl-5-methoxyphenol; BB0282781) for the indicated time periods. White arrowheads indicate forming membrane blebs. (D) Cells were treated as described in (C). ChIP experiments were performed using antibodies against TOPBP1. Enriched DNA was analyzed by qPCR with primer pair d1 specific for the rRNA gene promoter, as indicated in the scheme. Data are presented relative to input. Values represent mean ± sd from at least three independent replicates. **, *p* < 0.01 by unpaired t-test; n.S., not significant. (e) cells were treated as described in (C), and the proportion of cells displaying pronounced membrane blebbing was quantified. (F) Calcium dynamics were visualized using the fluorescent probe Fluo-4. Cells were loaded with Fluo-4, washed, and then exposed to 1 µM ionomycin for the indicated time periods. White arrowheads indicate forming membrane blebs. (G) Cells were treated as described in (f) and then fixed. ChIP experiments were performed using antibodies against TOPBP1. Enriched DNA was analyzed by qPCR with primer pair d1 specific for the rRNA gene promoter, as indicated in the scheme. Data are presented relative to input. Values represent mean ± sd from at least three independent replicates. **, *p* < 0.01 by unpaired t-test; n.S., not significant. (H) Cells were treated as described in (f), and the proportion of cells displaying pronounced membrane blebbing was quantified. (i) HeLa cells were treated with 300 µM olivetol or subjected to hypotonic stress for 30 min; with MG132 at 50 µM for up to 3 h or 500 nM for 72 h; with tunicamycin (Tm) at 20 µg/mL for 72 h; with CCT020312 (CCT) at 10 µM for 3 h or 1 µM for 24 h; or subjected to acute heat shock at 45°C for 30 min. ChIP experiments were then performed using antibodies against TOPBP1. Enriched DNA was analyzed by qPCR with primer pair d1 specific for the rRNA gene promoter, as indicated in the scheme. Data are presented relative to input. Values represent mean ± sd from at least three independent replicates. **, *p* < 0.01 by unpaired t-test; n.S., not significant. (J) HeLa cells were incubated with 10 mM methyl-β-cyclodextrin (MβCD) in complete culture medium for 1 h and, without removing MβCD, were subsequently treated with 300 µM olivetol for 30 min, subjected to hypotonic stress for 30 min, or treated with 70 µM VP16 for 30 min. Cells were then fixed and immunostained with antibodies against TOPBP1 (green). DNA was stained with DAPI (blue). (K) for membrane perturbation experiments, cells were pretreated with 1% Tween-20 in complete culture medium for 10 min, digitonin at 5 µg/mL in complete culture medium for 10 min, or 10 mM methyl-β-cyclodextrin (MβCD) in complete culture medium for 1 h. Cells were then treated with 300 µM olivetol for 30 min, subjected to hypotonic stress for 30 min, or treated with 70 µM VP16 for 30 min. ChIP experiments were subsequently performed using antibodies against TOPBP1. Enriched DNA was analyzed by qPCR with primer pair d1 specific for the rRNA gene promoter, as indicated in the scheme. Data are presented relative to input. Values represent mean ± sd from at least three independent replicates. **, *p* < 0.01 by unpaired t-test; n.S., not significant. (L) a schematic model illustrating the proposed mechanism is shown. Hypotonic stress or olivetol both disrupt plasma membrane lipid order and activate RNA polymerase II – dependent antisense transcription in rDNA. This may promote collision and interference between RNA polymerases I and II, resulting in R-loop formation and activation of the nucleolar DNA damage response (n-DDR). The sensor and signaling pathway connecting plasma membrane perturbation to the nucleolus remain unknown.

We first compared the kinetics of n-DDR activation with the kinetics of membrane blebbing. Recruitment of TOPBP1 to the rDNA promoter, measured by ChIP-qPCR, was rapid under both olivetol and hypotonic conditions and reached near-maximal levels within 5–10 min of treatment (Figure 5(A)). By contrast, membrane blebbing developed much more slowly and became most prominent only after ~60 min of exposure (Figure 5(B)). Consistently, nucleolar TOPBP1 relocalization was observed in nearly all cells following either olivetol or hypotonic stress, whereas the fraction of cells displaying overt membrane blebbing did not exceed ~65% even at later time points (Figure 5(B)). These differences in timing and penetrance indicate that membrane blebbing is not required for n-DDR activation.

This conclusion was further supported by the behavior of olivetol derivatives. As shown above, neither mono-methylated nor di-methylated olivetol analogs induced n-DDR (Figure 1(F)). However, their effects on membrane blebbing were clearly distinct: the mono-methylated analog triggered rapid and pronounced blebbing almost immediately after treatment (Figure 5(C-E); video-7), whereas the di-methylated analog caused little or no blebbing (Figure S5(A-C); video-8). Thus, membrane blebbing does not correlate with the ability of these compounds to activate nucleolar DDR.

We next examined whether calcium signaling could account for n-DDR induction. This also appeared unlikely. First, the increase in cytosolic calcium occurred substantially later than n-DDR activation (Figures 5(A) and 4(C,D)). Second, treatment with ionomycin, which induces robust calcium influx and pronounced membrane blebbing, failed to trigger n-DDR (Figures 5(F-H); video-9). Together, these observations indicate that neither calcium elevation nor secondary calcium-dependent membrane deformation is sufficient to trigger n-DDR. Given that both olivetol and hypotonic stress also induced an ER stress-like transcriptional program, we next tested whether classical ER stress is sufficient to activate the nucleolar response. Cells were therefore treated with several established ER stress inducers, including CCT020312, tunicamycin, MG132, and acute heat shock, under both short- and long-term conditions. None of these treatments induced nucleolar DDR (Figure 5(I)). Thus, although olivetol and hypotonic stress are both associated with an ER stress – like response, classical ER stress alone is not sufficient to account for n-DDR activation. Taken together, these results exclude membrane blebbing, calcium influx, and canonical ER stress as direct upstream triggers of olivetol- and hypotonic stress-induced nucleolar DDR.

Given our Laurdan measurements showing that both olivetol and hypotonic stress reduce plasma membrane lipid order, we next considered the possibility that n-DDR originates from a cholesterol-dependent sensor at the plasma membrane. To test this, we examined the effects of several membrane-active agents that perturb the plasma membrane through distinct mechanisms. Cells were treated with Tween-20, digitonin, or methyl-β-cyclodextrin (MβCD) prior to olivetol or hypotonic stress. Strikingly, neither Tween-20 nor digitonin had a detectable effect on n-DDR induced by either stimulus (Figure 5(K)). By contrast, MβCD almost completely abolished nucleolar DDR under both conditions (Figure 5(J,K)). Importantly, MβCD did not suppress the nucleolar DDR induced by the genotoxic agent etoposide (Figure 5(K)), indicating that its effect is specific to the non-genotoxic pathway activated by olivetol and hypotonic stress. These findings demonstrate that not all forms of membrane perturbation are equivalent with respect to n-DDR activation. Rather, the response depends specifically on membrane organization maintained by cholesterol, pointing to the involvement of a cholesterol-sensitive plasma membrane sensor or membrane state upstream of nucleolar DDR. In contrast, classical genotoxic n-DDR triggered by etoposide remains insensitive to cholesterol depletion, indicating that the non-genotoxic pathway activated by olivetol and hypotonic stress is mechanistically distinct.

Together, our data support a model in which olivetol and hypotonic stress activate nucleolar DDR through a shared, non-genotoxic pathway that originates at the plasma membrane and depends on cholesterol-sensitive membrane organization. This pathway is independent of membrane blebbing, delayed calcium influx, and canonical proteotoxic ER stress, and therefore defines a distinct form of membrane-to-nucleolus signaling (Figure 5(L)).

## Discussion

Olivetol is a short-chain member of the alkylresorcinol family, a group of phenolic lipids with diverse biological activities, including antioxidant, hypolipidemic, and anti-obesity effects [43]. Despite growing pharmacological interest, its molecular mechanism of action has remained unclear. Here we show that olivetol induces a non-genotoxic nucleolar DNA damage response (n-DDR) characterized by γH2AX accumulation at ribosomal DNA (rDNA), recruitment of TOPBP1 to Treacle, repression of RNA polymerase I transcription, and rapid reversibility after stress removal [17,18,21–23]. In these respects, olivetol closely phenocopies the selective nucleolar response triggered by hypotonic stress, while differing from classical genotoxic stimuli by the absence of detectable DNA damage elsewhere in the genome and by replication independence.

Our data further indicate that, under both olivetol and hypotonic conditions, n-DDR depends on R-loop accumulation within rDNA and on simultaneous participation of RNA polymerase I and RNA polymerase II. A parsimonious model is that antisense Pol II transcription within the rRNA coding region, likely related to PAPAS [28,29], proceeds while Pol I continues sense transcription of pre-rRNA, thereby generating transcriptional interference within the same rDNA repeat and promoting R-loop stabilization. Consistent with this idea, R-loops accumulated predominantly in the coding region, most strongly in the 28S rRNA region, and RNase H1 overexpression significantly reduced TOPBP1 recruitment. At the same time, RNase H1 only partially suppressed TOPBP1 accumulation, indicating that R-loops alone are unlikely to fully explain n-DDR activation. Additional topological stress generated by convergent transcription may also contribute. Opposing transcription complexes are expected to generate torsional strain, local DNA unwinding, and transient single-stranded DNA, all of which could facilitate ATR/TOPBP1-dependent signaling [44–46]. The strong enrichment of R-loops in the 28S region further suggests that local sequence features, including high GC content and a propensity for non-B DNA formation, may stabilize these transcription-associated structures [47–49].

Notably, not all conditions that induce antisense rDNA transcription appear sufficient to trigger n-DDR. Heat shock [27] and serum starvation [29], for example, are known to activate PAPAS, yet in our hands neither condition produced TOPBP1 recruitment or γH2AX accumulation at rDNA. A likely explanation is that these stresses strongly repress Pol I transcription [50,51], thereby preventing simultaneous sense and antisense elongation within the coding region. In the absence of ongoing Pol I transcription, antisense transcription alone may be insufficient to generate the transcriptional conflict required for full n-DDR activation.

Beyond nucleolar DDR, olivetol produced a broad membrane-associated phenotype that closely resembled the cellular response to hypotonic stress. Both treatments caused calcium redistribution, plasma membrane blebbing, reduced membrane lipid order, and transcriptional changes consistent with ER stress, while at higher concentrations olivetol also directly compromised membrane integrity. This membrane-associated phenotype is particularly relevant in light of the pharmacological activities previously attributed to olivetol. Its anti-obesogenic and lipid-lowering effects have been linked to CB1-related activity and inhibition of HMG-CoA reductase [52], and in silico studies have suggested additional interactions with metabolic receptors such as cholecystokinin and GLP-1 receptors [4]. However, our data suggest that at least part of olivetol action is better explained by direct perturbation of membrane structure than by classical receptor-mediated signaling. Notably, although olivetol displaces the CB1 agonist [3 H]CP55,940, it does not measurably affect canonical CB1-dependent cAMP signaling [52], raising the possibility that its apparent receptor activity reflects indirect effects on membrane organization, as proposed for other highly lipophilic compounds [53]. Together, these observations support the view that olivetol acts primarily as a membrane-active molecule, with receptor effects, if any, being secondary to a more general membrane-level mechanism.

At the same time, our data argue that neither delayed calcium influx, nor membrane blebbing, nor classical proteotoxic ER stress is sufficient to account for n-DDR activation. Thus, although olivetol and hypotonic stress share multiple membrane-associated and ER stress-like phenotypes, these responses are more likely to represent parallel consequences of a common upstream membrane disturbance than direct triggers of the nucleolar pathway.

An important implication of our data is therefore that the upstream signal linking olivetol and hypotonic stress to nucleolar DDR is likely encoded at the level of plasma membrane organization. This is strongly supported by the selective effect of methyl-β-cyclodextrin: because MβCD disrupts cholesterol-dependent membrane organization rather than simply permeabilizing the membrane [54–56], our results argue that the critical upstream event is not generic membrane damage, but perturbation of a cholesterol-sensitive membrane state.

One plausible candidate for such a membrane sensor is the caveolar system. Caveolae are cholesterol-rich membrane domains that respond to hypotonic and mechanical stress by flattening or disassembling, and recent work suggests that caveolar deformation can influence nuclear transcription through redistribution of cavin-1 [57,58]. Notably, PTRF/cavin-1 has also been implicated in regulation of ribosomal RNA transcription under metabolic challenge, independently of its structural role in caveolae [59–61]. These observations make the caveolae-cavin axis an attractive conceptual link between plasma membrane perturbation and the highly selective nucleolar response described here. It is therefore tempting to speculate that olivetol and hypotonic stress converge on a common cholesterol-dependent membrane signal that modulates transcription within rDNA, for example by facilitating antisense Pol II transcription and thereby promoting transcriptional conflict with Pol I and stabilization of R-loops.

Thus, while the molecular nature of the membrane-to-nucleolus pathway remains to be defined, our data identify a previously unrecognized cholesterol-sensitive membrane-associated mechanism capable of activating nucleolar DDR in the absence of detectable DNA breaks. More broadly, our results provide a conceptual framework for understanding how membrane-active small molecules can influence nuclear stress signaling and nucleolar function.

## Supplementary Material

Supplementary data.docx

## Data Availability

All video files are available in the Zenodo repository under DOI: 10.5281/zenodo.19130191. Sequencing raw read files are available in a ENA repository: PRJEB101390 (ERP182796).
