# Supplementary material for: Olivetol induces a non-genotoxic nucleolar DNA damage response via membrane-dependent stress signaling
Source: Nucleus. 2026 May 18;17(1):2672818. doi: 10.1080/19491034.2026.2672818 (PMC13185464; doi:10.1080/19491034.2026.2672818)
Supplement: Supplementary data.docx [file KNCL_A_2672818_SM6013.docx]

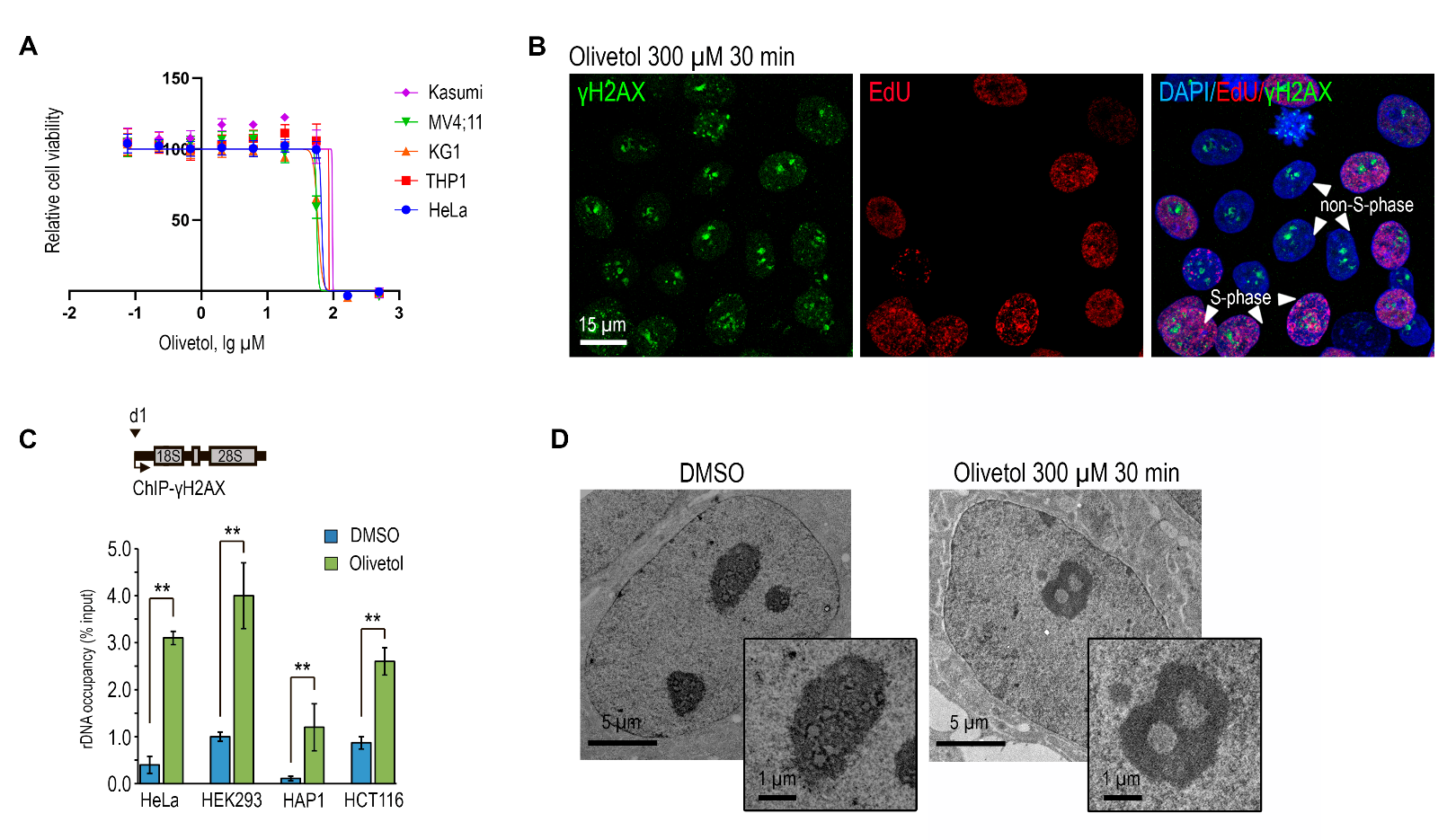


**Figure S1. Olivetol induces nucleolar γH2AX accumulation in multiple cell lines and inhibits nucleolar transcription. (A)** Dose–response curves for Kasumi, MV4;11, KG1, THP1, and HeLa cells treated with increasing concentrations of olivetol for 5 days. Data are presented as mean ± SD (n = 3 biologically independent experiments). **(B)** For 5-ethynyl-2′-deoxyuridine (EdU) incorporation, HeLa cells were pulse-labeled with 10 µM EdU in the presence of 300 µM olivetol for 30 min at 37 °C. Cells were stained for γH2AX (green). EdU was detected by click chemistry (red). DNA was stained with DAPI (blue). **(C)** HeLa, HEK293, HAP1, and HCT116 cells were treated with 300 µM olivetol for 30 min or with DMSO as a negative control, followed by ChIP using antibodies against γH2AX. Enriched DNA was analyzed by qPCR with primer pair d1 specific for the rRNA gene promoter, as indicated in the scheme. Data are presented relative to input. Values represent mean ± SD from at least three independent experiments. **, p < 0.01 by unpaired t-test; n.s., not significant. **(D)** HeLa cells were treated with 300 µM olivetol for 30 min and analyzed by transmission electron microscopy (TEM).


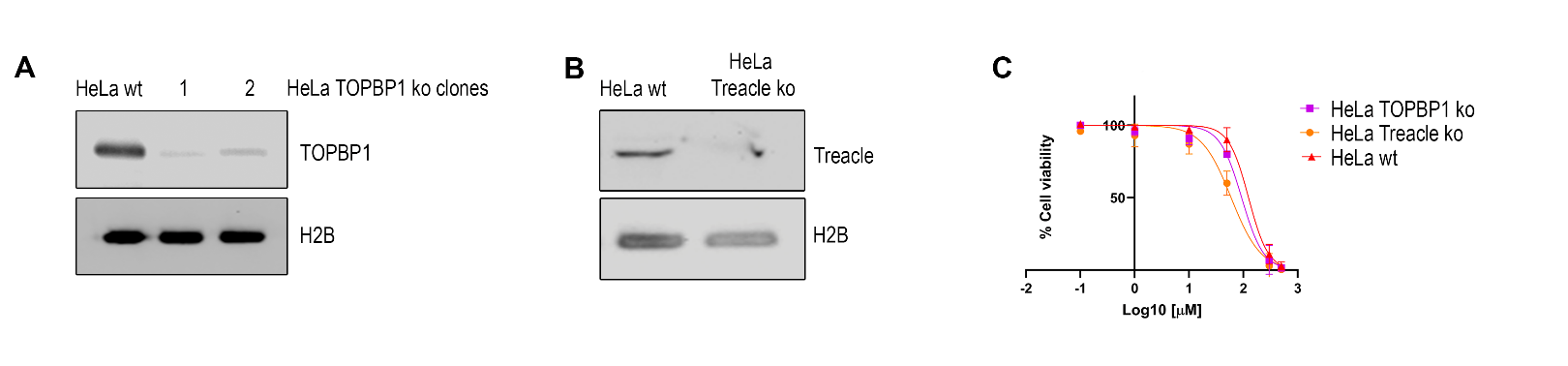
**Figure S2. Treacle and TOPBP1 knockout validation and olivetol dose–response analysis in HeLa cells. (A)** HeLa cells were depleted of TOPBP1 using CRISPR/Cas9 technology. Knockout efficiency was analyzed by western blotting. Histone H2B was used as a loading control. **(B)** HeLa cells were depleted of TCOF1 (Treacle) using CRISPR/Cas9 technology. Knockout efficiency was analyzed by western blotting. Histone H2B was used as a loading control. **(C)** Dose–response curves for HeLa wild-type (HeLa wt) cells, TOPBP1 knockout cells, and Treacle knockout cells treated with increasing concentrations of olivetol for 5 days. Data are presented as mean ± SD (n = 3 biologically independent experiments).


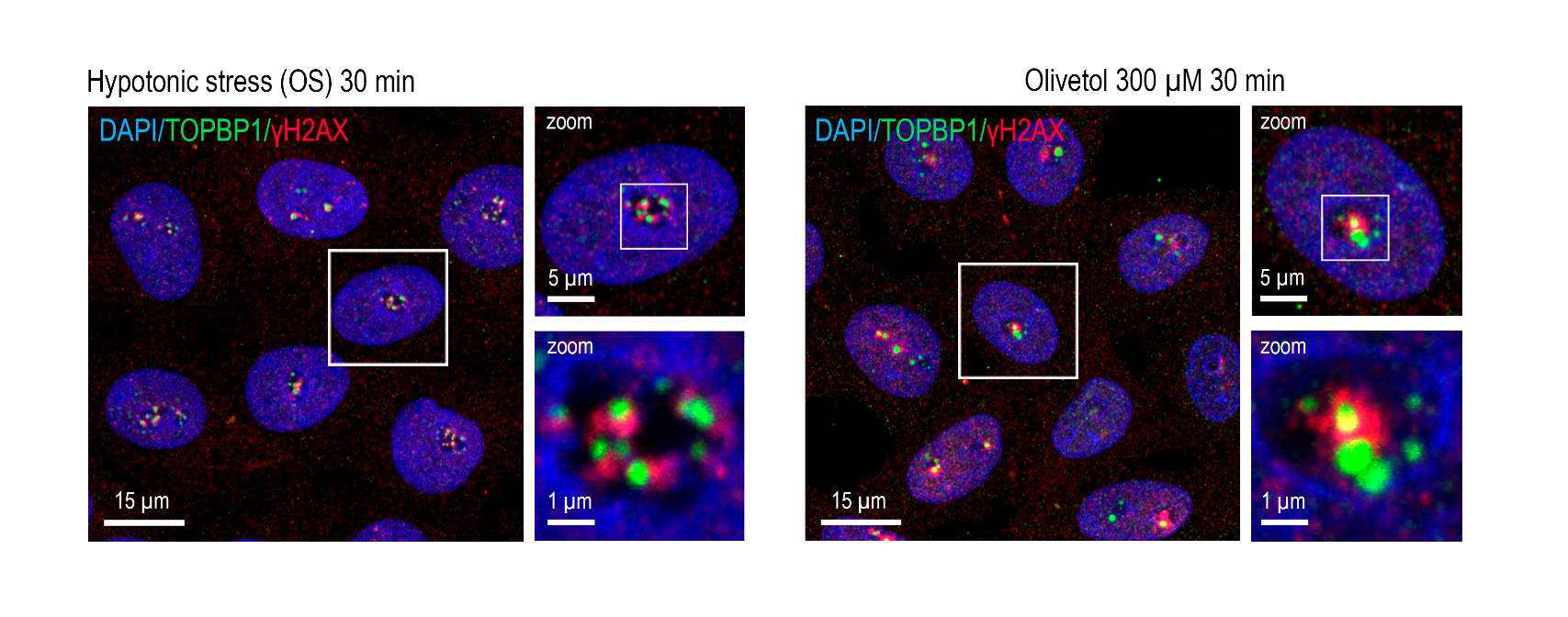
**Figure S3. Olivetol and hypotonic stress induce nucleolar colocalization of TOPBP1 and γH2AX.** HeLa cells were treated with 300 µM olivetol or subjected to hypotonic stress (OS) for 30 min. Cells were co-immunostained for TOPBP1 (green) and γH2AX (red). DNA was stained with DAPI (blue).


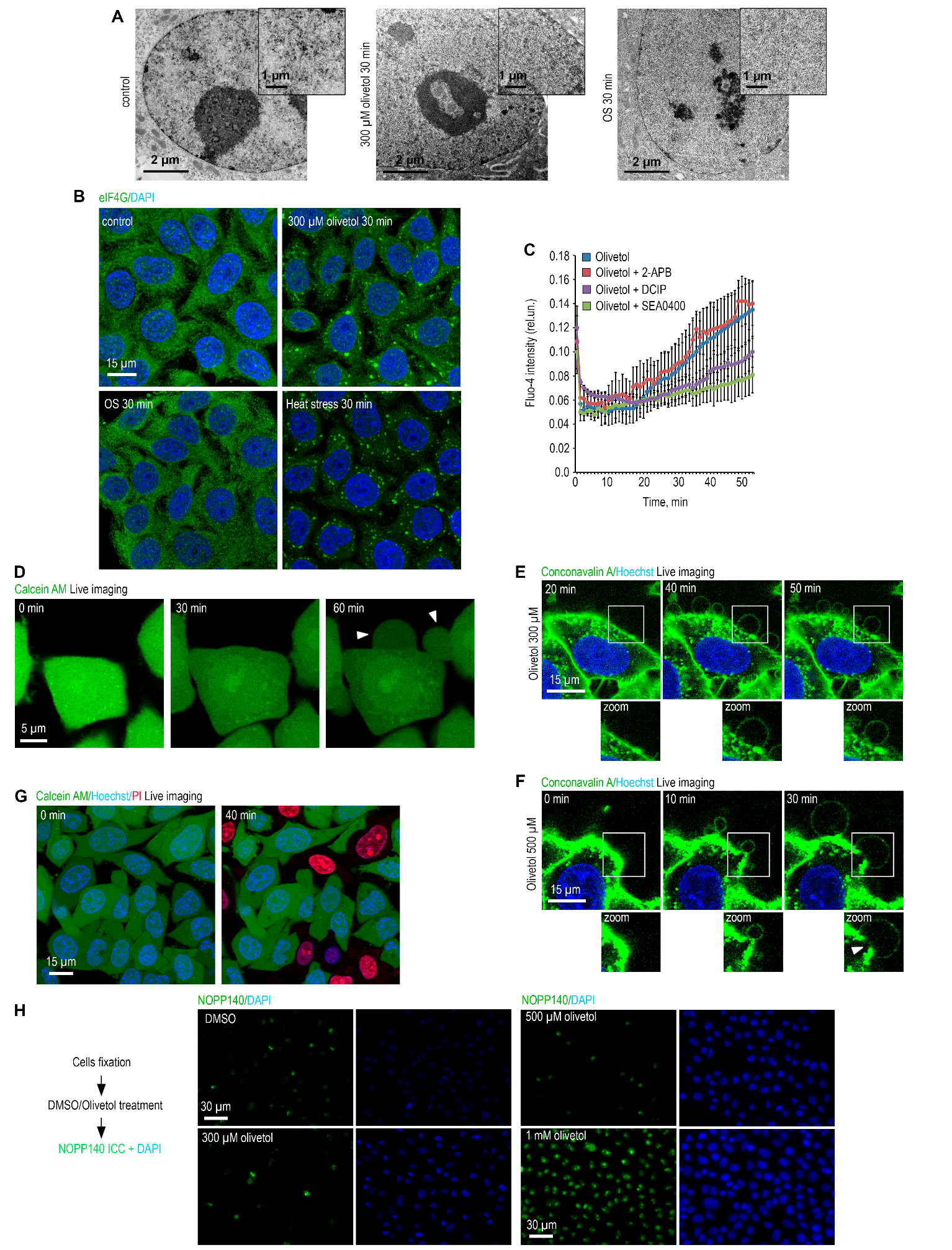


**Figure S4.** **Olivetol and hypotonic stress induce membrane blebbing and alter plasma membrane organization. (A)** HeLa cells were treated with 300 µM olivetol or subjected to hypotonic stress (OS) for 30 min and analyzed by transmission electron microscopy (TEM). **(B)** HeLa cells were treated with 300 µM olivetol for 30 min, subjected to osmotic stress (OS) for 30 min, or exposed to heat shock at 45 °C for 30 min as a positive control for stress granule formation. Cells were then fixed and immunostained with antibodies against eIF4G (green). DNA was stained with DAPI (blue). **(C)** Calcium dynamics were visualized using the fluorescent probe Fluo-4. Cells were loaded with Fluo-4, washed, and then exposed to 300 µM olivetol or olivetol in the presence of channel inhibitors, 50 µM 2-APB, 10 µM DCPIB, or 2 µM SEA0400, for the indicated time periods. Fluorescence intensity was quantified in each frame using CellProfiler across more than 50 individual cells. Data are presented as mean ± SD. **(D)** HeLa cells were loaded with Calcein AM and then subjected to hypotonic stress for the indicated time periods. Time-lapse imaging was performed at a rate of one frame per minute. Image processing was carried out using ImageJ. White arrowheads indicate forming membrane blebs. **(E)** For live-cell imaging of membrane blebbing, cells were incubated with CF488-conjugated Concanavalin A and Hoechst 33342, followed by exposure to 300 µM olivetol for the indicated time periods. Time-lapse imaging was performed at a rate of one frame per minute. Image processing was carried out using ImageJ. **(F)** Cells were treated with 500 µM olivetol and analyzed as described in (E). **(G)** Cells were incubated with Calcein AM, propidium iodide (PI), and Hoechst 33342, followed by exposure to 500 µM olivetol for the indicated time periods. Time-lapse imaging was performed at a rate of one frame per minute. Image processing and quantitative analysis were carried out using ImageJ. **(H)** HeLa cells were fixed with 1% formaldehyde and then treated with olivetol at the indicated concentrations for 30 min. Cells were subsequently immunostained with antibodies against NOPP140, and DNA was stained with DAPI.


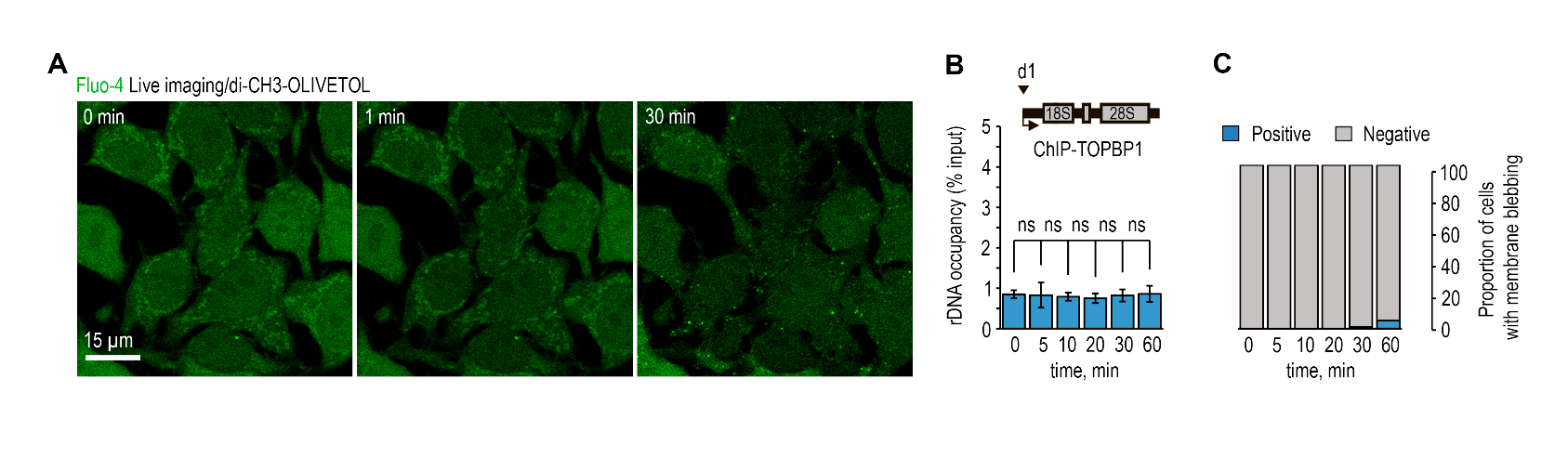
**Figure S5. Di-CH3-olivetol fails to induce membrane blebbing and TOPBP1 recruitment to rDNA. (A)** Calcium dynamics were visualized using the fluorescent probe Fluo-4. Cells were loaded with Fluo-4, washed, and then exposed to 300 µM di-CH3-olivetol (1-butyl-3,5-dimethoxybenzene; BB0282782) for the indicated time periods. White arrowheads indicate forming membrane blebs. **(B)** Cells were treated as described in (A). ChIP experiments were performed using antibodies against TOPBP1. Enriched DNA was analyzed by qPCR with primer pair d1 specific for the rRNA gene promoter, as indicated in the scheme. Data are presented relative to input. Values represent mean ± SD from at least three independent replicates. **, p < 0.01 by unpaired t-test; n.s., not significant. **(C)** Cells were treated as described in (A), and the proportion of cells displaying pronounced membrane blebbing was quantified.

|  | forward (5'-3') | reverse (5'-3') |
| --- | --- | --- |
| sgRNA1 TCOF | CACCGAAGTAGCTCCCGCCGCTTCC | AAACGGAAGCGGCGGGAGCTACTTC |
| sgRNA2 TCOF | CACCGGGGGTCGCGGGTATGGCCG | AAACCGGCCATACCCGCGACCCCC |
| sgRNA1 TOPBP1 | САССGAAACTGGATGTTCGGCTCTT | AAACAAGAGCCGAACATCCAGTTTC |
| sgRNA1 TOPBP2 | САССGATATATCTTTGCGGTTTTAG | AAACCTAAAACCGCAAAGATATATC |

**Supplementary Table S1.** List of primers used for knockdown and knockout. Added restrictive sites are highlighted in red.

**Supplementary Table S2.** List of antibodies used in this study.

| Antibody | anti-Treacle/TCOF1 (Mouse monoclonal) | Santa Cruz Biotechnology | sc-374536, RRID:AB_10987865 | ICC (1:500), WB (1: 5000), |
| --- | --- | --- | --- | --- |
| Antibody | anti-Treacle/TCOF1 (Rabbit polyclonal) | Sigma-Aldrich | HPA038237 | ICC (1:500) |
| Antibody | anti-RPA194 (Mouse monoclonal) | Santa Cruz Biotechnology | sc-48385, RRID:AB_675814 | ICC (1:50), |
| Antibody | anti-UBF1 (Rabbit polyclonal) | ThermoFisher | PA5-82245, RRID:AB_2789405 | ICC (1:200), |
| Antibody | anti-Nucleolin (Rabbit Monoclonal) | Cell Signaling | 14574, RRID:AB_2798519 | ICC (1:500) |
| Antibody | anti-TOPBP1 (Mouse Monoclonal) | Santa Cruz Biotechnology | sc-271043, RRID:AB_10610636 | ICC (1:200), ChIP-qPCR,  WB (1:2500) |
| Antibody | anti-NOPP140  (Mouse Monoclonal) | Santa Cruz Biotechnology | sc-374033,  RRID:AB_10917069 | ICC (1:500) |
| Antibody | anti-Ki67 (Rabbit Monoclonal ) | Cell Signaling | #9129, RRID:AB_2687446 | ICC (1:500) |
| Antibody | anti-γH2AX (Ser139) (Mouse Monoclonal) | Millipore | 05-636, RRID:AB_309864 | ICC (1:200)  ChIP-qPCR |
| Antibody | Anti-R-loop S9.6, (Mouse Monoclonal) | Millipore | #MABE1095,  RRID:AB_2861387 | DRIP-ChIP-qPCR |
| Antibody | Anti-RNA polymerase II CTD repeat YSPTSPS (phospho S2) (Rabbit polyclonal) | Abcam | ab5095  RRID:AB_304749 | ChIP-seq |
| Antibody | anti-GAPDH, (Rabbit polyclonal) | Abcam | ab9485, RRID:AB_307275 | WB |
| Antibody | anti-eIF4G | Cell Signaling | #2498, RRID:AB_2096025 | ICC (1:200) |
| Antibody | Polyclonal Goat anti-mouse Alexa Fluor Plus 488 | Invitrogene | A32723, RRID:AB_2633275 | ICC (1:200) |
| Antibody | Polyclonal Goat anti-mouse Alexa Fluor Plus 594 | Invitrogene | A32742, RRID:AB_2762825 | ICC (1:200) |
| Antibody | Polyclonal Goat anti-rabbit CF488A | Biotium | #20012, RRID:AB_10853801 | ICC (1:200) |
| Antibody | Polyclonal Goat anti-rabbit CF594 | Biotium | #20112, RRID:AB_10559190 | ICC (1:200) |

**Supplementary Table S3.** List of primers used for ChIP-qPCR.

|  | forward (5'-3') | reverse (5'-3') |
| --- | --- | --- |
| ChIP-qPCR d1 rDNA | GGTATATCTTTCGCTCCGAGTC | ACAGGTCGCCAGAGGACAG |
| ChIP-qPCR ALU | ACCATCCCGGCTAAAACGGTGA | GCGATCTCGGCTCACTG |

**Supplementary Table S4**. List of primers used for DRIP-ChIP-qPCR.

|  | forward (5'-3') | reverse (5'-3') |
| --- | --- | --- |
| DRIP-ChIP primer 1 | TGGCCCTTACGCTCAGAATG | GAGTTTGGCTCTTGCTGCC |
| DRIP-ChIP primer 2 | GCTTCTCCCTCGACTGTCTC | GGCTCGGTCACAGATCACTT |
| DRIP-ChIP primer 3 | AGTGAAGACAACTCACGCCC | CTGCAAACGGGAACACGA |
| DRIP-ChIP primer 4 | AATGGCCTTAGCCCTGGTG | GTCCAGAGACGAGAGACCGA |
| DRIP-ChIP primer 5 | GGCCCGATTGTTCTTCTCCT | CTGCCATCTGTCAAACCCGA |
| DRIP-ChIP primer 6 | GACAACGTGTATCTCTGCATT | ACACAGACACCTACATCTATCA |
| DRIP-ChIP primer 7 | GGTATATCTTTCGCTCCGAGTC | ACAGGTCGCCAGAGGACAG |
| DRIP-ChIP primer 8 | GCCTTCTCTAGCGATCTGAGAG | CCATAACGGAGGCAGAGACA |
| DRIP-ChIP primer 9 | CAGCGTGTGCCTACCCTAC | TCCCTCGTTCATGGGGAATAA |
| DRIP-ChIP primer 10 | GGTTGCTTGGGAATGCAG | CTTGTTGACTATCGGTCTCGTG |
| DRIP-ChIP primer 11 | TCCCTCCGAAGTTTCCCTCA | CGGCCCCAAGACCTCTAATC |
| DRIP-ChIP primer 12 | TGGCGCTAAACCATTCGTAG | GTCGAGGGCTGACTTTCAATAG |
| DRIP-ChIP primer 13 | CGCCTGGTCTTCTGTCTCTG | CGTAAGCTGGAGTGGAAGTGT |
| DRIP-ChIP primer 14 | TTGACGTACAGGGTGGACTG | GGCATCCTAGGTGACATTTCC |

**Supplementary Table S5.** Analytical data for 1-Butyl-3,5-dimethoxybenzene (BB 0282782).

**Supplementary Table S6.** Analytical data for 3-Butyl-5-methoxyphenol (BB 0282781).
